# Global dynamics and computational modeling approach for analyzing and controlling of alcohol addiction using a novel fractional and fractal–fractional modeling approach

**DOI:** 10.1038/s41598-024-54578-9

**Published:** 2024-03-01

**Authors:** Shuo Li, Samreen, Saif Ullah, Muhammad Bilal Riaz, Fuad A. Awwad, Shewafera Wondimagegnhu Teklu

**Affiliations:** 1 School of Mathematics and Data Sciences, Changji University, Changji, 831100 Xinjiang People’s Republic of China; 2 Department of Mathematics, Abdul Wali Khan University Mardan, Mardan, Khyber Pakhtunkhwa, Pakistan; 3 Department of Mathematics, University of Peshawar, Peshawar, Khyber Pakhtunkhwa Pakistan; 4 IT4Innovations, VSB -Technical University of Ostrava, Ostrava, Czech Republic; 5 Department of Quantitative analysis, College of Business Administration, King Saud University, P.O. Box 71115, Riyadh, 11587 Saudi Arabia; 6 Department of Mathematics, College of Natural and Computational Scieces, Debre Berhan University, 445 Debre Berhan, Ethiopia; 7 Department of Computer Science and Mathematics, Lebanese American University, Byblos, Lebanon

**Keywords:** Alcohol addiction model, Fractal–fractional Caputo operator, Ulam–Hyers stability, Existence and uniqueness, Simulation, Computational biology and bioinformatics, Mathematics and computing

## Abstract

In recent years, alcohol addiction has become a major public health concern and a global threat due to its potential negative health and social impacts. Beyond the health consequences, the detrimental consumption of alcohol results in substantial social and economic burdens on both individuals and society as a whole. Therefore, a proper understanding and effective control of the spread of alcohol addictive behavior has become an appealing global issue to be solved. In this study, we develop a new mathematical model of alcohol addiction with treatment class. We analyze the dynamics of the alcohol addiction model for the first time using advanced operators known as fractal–fractional operators, which incorporate two distinct fractal and fractional orders with the well-known Caputo derivative based on power law kernels. The existence and uniqueness of the newly developed fractal–fractional alcohol addiction model are shown using the Picard–Lindelöf and fixed point theories. Initially, a comprehensive qualitative analysis of the alcohol addiction fractional model is presented. The possible equilibria of the model and the threshold parameter called the reproduction number are evaluated theoretically and numerically. The boundedness and biologically feasible region for the model are derived. To assess the stability of the proposed model, the Ulam–Hyers coupled with the Ulam–Hyers–Rassias stability criteria are employed. Moreover, utilizing effecting numerical schemes, the models are solved numerically and a detailed simulation and discussion are presented. The model global dynamics are shown graphically for various values of fractional and fractal dimensions. The present study aims to provide valuable insights for the understanding the dynamics and control of alcohol addiction within a community.

## Introduction

Alcohol addiction, also known as alcoholism is a chronic and frequently progressive disease characterized by an individual’s dependence on and misuse of alcohol. Alcohol addiction can lead to various negative consequences, such the individual health problems, relationship issues, social status, impaired judgment, accidents, loss of interest, and overall quality of life. People with alcohol addiction often find it difficult to control their alcohol consumption and may experience strong cravings for alcohol. The key features of alcohol addiction include compulsive drinking, loss of control, physical dependence, continued use despite harm, etc. Misuse of alcohol is a recognized causal factor in the development of over 200 diseases, injuries, and various other health issues. Drinking alcohol addiction is linked to an increased risk of experiencing health issues, including mental and behavioral disorders like alcohol dependence, as well as significant non-communicable illnesses such as liver cirrhosis, certain types of cancer, and cardiovascular diseases^1^. According to the World Health Organization (WHO) statistics, globally, more than 3 million deaths occur each year due to the harmful consumption of alcohol leading to roughly 5.3% of all deaths worldwide. Furthermore, alcohol contributes significantly to the global burden of disease and injury, comprising approximately 5.1% of this burden^1,2^. Treatment for people with alcohol addiction often involves a combination of behavioral therapy, support groups, counseling, and in some cases, medication. Treatment aims to help individuals achieve and maintain sobriety, improve their overall well-being, and develop healthy coping strategies. Early intervention and support from loved ones can play a crucial role in recovery from alcohol addiction. Recovery from alcohol addiction is possible with the proper support and commitment to change.

Analyzing the dynamical aspect of any complex real-world problem is necessary before setting effective preventive controlling interventions. Various tools have been utilized for this purpose. Mathematical modeling is a useful and interdisciplinary approach that helps researchers and decision-makers gain insights into complex problems, make informed choices, predict future and current behavior, and set some effective interventions. It plays a crucial role in scientific research, engineering design, policy analysis, and various other applications. These models utilize mathematical equations, algorithms, and representations to simulate, analyze, and predict the behavior of complex systems. The transmission models are usually constructed with the help of various differential derivatives, for instance with partial, stochastic, ordinary, and fractional order in nature^3–7^. In^8^ a discrete-time transmission model has been formulated addressing the dynamics and control of alcohol addiction illness with physical and psychological complications. A similar study of a novel alcohol drinking model comprising liver complication was studied in^9^. A classical deterministic modeling approach has been adopted to explore insights into alcohol addiction behavior with different susceptibilities^10^. A comprehensive mathematical analysis to understand the role of the misuse of alcohol on gonorrhea infection has been conducted in^11^. Moreover, the authors developed some effective and optimal interventions for the mitigation of the aforementioned infection.

Fractional differential equations generalize the classical integer order systems. The classical integer order system can be considered a special case of the fractional system by setting the fractional order to an integer value. In recent years, mathematical modeling based on fractional and fractal–fractional operators has gained significant interest from researchers worldwide. Fractional and fractal–fractional mathematical modeling is a valuable tool for describing and analyzing complex systems that exhibit memory effects, anomalous behaviors, and self-similar behavior^12,13^. These mathematical models employ fractional or fractal–fractional derivatives and integrals instead of classical integer-order derivatives and integrals to capture the complex behavior observed in infectious and non-infectious diseases more accurately^14^. Additionally, a system with such operators provides a greater degree of accuracy and a better fit to the real data in order to predict future dynamics of the problem^15,16^. Specifically, in epidemiology, individuals may carry immunity or susceptibility to diseases for extended periods, which is better described by fractional-order models^17^. The commonly recognized fractional operators employed in existing literature include the Caputo operator^18^, the Caputo–Fabrizio operator (CF)^19^, and the Atangana-Baleanu operators (ABC)^20^.

The application of these fractional operators can be found extensively in almost all fields of science. The implementation of ABC fractional modeling approach for a time-variable predator-pray model was presented in^21^. Controllability outcomes concerning fractional differential equations of neutral type with ABC derivatives were established in the work by Bedi et al.^22^. A fractional compartmental modeling approach in the Caputo sense was adopted in^23^ addressing the role of alcohol-addictive behavior leading to road accidents. The potential application of fractional derivative in exporting the dynamics of Zika viral epidemic was studied in^24^. Moreover, in^23^ the authors demonstrated the impact of memory effects on the model dynamics. A novel non-integer order compartmental model with the Caputo derivative describing the dynamics of giving up smoking was presented in^25^. The analysis of a transmission model addressing social media addiction was studied in^26^. The application of fractional modeling and optimal control theory to the online game addiction model has been developed recently in^27^. A fractional mathematical modeling approach for describing the dynamical aspect and preventive measure of alcoholism can be found in^28^. The authors in^28^ a comprehensive qualitative and qualitative analysis of the propped model in fractional case. The modeling technique utilizing innovative fractal–fractional operators gained much interest after introducing these operators in^29^. In recent times, numerous researchers have effectively applied these fresh concepts to address a wide array of scientific challenges, including applications in epidemics^30–32^.

This study presents the formulation of a new mathematical model to explore the dynamics and control of alcohol addiction, one of the leading global health challenges. The treatment class for the heavy drinkers is considered in the model with the assumption that some of the heavy drinkers quit the addictive behavior by passing through proper medications, and other psychological and social interventions. The novel fractal–fractional operator based on power-law kernel is used to develop the model. To the best of our knowledge, we attempted for the first to analyze the dynamical aspects of the alcohol addiction model using such modeling techniques. The existence of the newly formulated fractal–fractional alcohol addiction model is presented using the Picard-Lindel$\ddot{o}$f theorem. Furthermore, we explore the influence of variation in fractal and fractional orders as well as transmission coefficients on the persistence and potential eradication of alcohol addiction using efficient numerical schemes. The study is performed in the following sections: After a brief introduction in the first section, we provide basic definitions in ““Preliminaries” section. The model formulation in the classical case is presented in “Model construction in classical sense” section. In “Fractional extension of the alcohol addiction model” section, we extend the alcohol addiction model to the fractional case and conduct a comprehensive qualitative analysis. “Numerical scheme and simulation discussion” Section includes the numerical solutions, dynamic visuals, and discussions. Additionally, in “Alcohol addiction model in fractal–fractional perspective” section, we introduce the construction of the fractal–fractional alcohol addiction model with a concise theoretical and numerical analysis. The concluding remarks of our work are presented in “Conclusion” section.

## Preliminaries

We recall some necessary definitions from the literature^18,29^.

### Definition 1

Consider a function $\texttt {V}(t)$ in the space $C^m$, where *m* is a natural number and $m-1 < \vartheta \le m$ represents the fractional order (FO), the Caputo derivative is defined as follows:

$$\begin{aligned} {}^CD^{\vartheta }_{t}(\texttt {V}(t))= \frac{1}{\Gamma (m-\vartheta )}\int \nolimits _0^t (t-s)^{m-\vartheta -1} \frac{d^m\texttt {V}(s)}{ds^m}ds. \end{aligned}$$

${}^CD^{\vartheta }_{t}(\texttt {V}(t))$ approaches to integer case when $\vartheta \rightarrow 1$.

### Definition 2

For the system

1 $$\begin{aligned} {}^CD^{\vartheta }_{t}\nu (t)=f(t,\nu (t)), \;\; \vartheta \in (0,1), \end{aligned}$$

if $f(t,\nu ^*)=0$, then the point $\nu ^*$ is called an equilibrium point.

Assume that the function $\texttt {V}(t)$ exhibits continuous fractal differentiability over the interval $(c_1, c_2)$. We consider the following fractal–fractional derivative and integral in the Caputo case, as referenced in^29^:

### Definition 3

The fractal–fractional operator based on the power-law is stated as follows:

2 $$\begin{aligned} {}^{{FFP}}D^{\vartheta , \zeta }_{0, t} \Big (\texttt {V}(t)\Big )= & {} \frac{1}{\Gamma (m-\vartheta )}\frac{d}{dt^{\zeta }}\int \nolimits ^t_0{(t-s)}^{m-\vartheta -1}{} \texttt {V}(s)ds, \end{aligned}$$

where, $m-1<\vartheta , \zeta \le m$ $\in \mathbb {N}$ are the fractional and fractal dimensions respectively, and $\frac{d\texttt {V}(s)}{ds^{\zeta }}=\lim _{t\rightarrow s}\frac{\texttt {V}(t)-\texttt {V}(s)}{t^{\zeta }-s^{\zeta }}.$

### Definition 4

The fractal–fractional integral operator of $\texttt {V}(t)$ with order $\vartheta$ is given through the following formula:

3 $$\begin{aligned} {}^{{FFP}}I^{\vartheta }_{0, t} \Big (\texttt {V}(t)\Big )= & {} \frac{\zeta }{\Gamma (\vartheta )}\int \nolimits ^t_0{(t-s)^{\vartheta -1}s^{\zeta -1}}{} \texttt {V}(s)ds. \end{aligned}$$

## Model construction in classical sense

The construction process for the alcohol behavior model is briefly described in this section. The total population is categorized into seven groups according to their epidemiological status. At the time instance *t*, the potential drinkers are denoted by $\mathcal {P}(t)$; occasional or moderate alcohol drinkers are $\mathcal {M}(t)$; Heavy alcohol drinkers are $\mathcal {H}(t)$; under treatment drinkers are $\mathcal {T}(t)$; violence creating alcohol drinkers are $\mathcal {V}(t)$; heavy alcohol drinkers who cause traffic accident are $\mathcal {A}(t)$ and the recovered or quitter drinkers are placed in $\mathcal {Q}(t)$ class.

The class $\mathcal {P}(t)$ includes individuals aged over adolescence and adulthood and has the potential to become drinkers. The population in $\mathcal {P}(t)$ are recruited at the rate $\Lambda$ and reduces due to contacts with the individuals in $\mathcal {M}(t)$ and $\mathcal {H}(t)$ compartments at the rates $\beta _{1}$ an $\beta _{2}$ respectively. The potential drinkers are further decreased because of natural death at a rate $\nu$.

The class of moderate or occasional drinkers includes those individuals who can manage their alcohol consumption during some social gatherings, or whose consumption is hidden from their social circle. These drinkers do not experience any social issues or negative effects from alcohol use. These drinkers do not regularly consider drinking or frequently feel the desire to drink, which is one of their traits. They do not frequently fight, lose their cool, or act violently. The potential drinkers move to the occasional drinkers at a rate $\beta _{1}$ resulting in an increase in the population in this compartment. Furthermore, the interaction of $\mathcal {P}(t)$ with $\mathcal {H}(t)$ at a rate $\beta _{2}$ results in an increase in this compartment. It is diminished by the natural death rate $\nu$ and due to the transition to heavy drinkers at a rate $\psi$.

The class of heavy drinkers includes individuals who exhibit severe alcohol addiction and can potentially pass on the addiction to individuals of the class $\mathcal {P}(t)$ when they interact with heavy drinkers^33,34^. A person with alcoholism has trouble controlling or restricting their harmful use of alcohol. The moderate drinkers become heavy drinkers and join $\mathcal {H}$ class by the rate $\psi$. The class of heavy drinkers decreased because of the transition of individuals from $\mathcal {H}(t)$ to $\mathcal {A}(t)$, $\mathcal {V}(t)$, $\mathcal {Q}(t)$ and $\mathcal {T}(t)$ groups at the rates $\theta _{2}$, $\theta _{1}$, $\theta _{3}$ and $\phi$ respectively. In addition, this class is reduced by the alcohol-induced death rate $\delta _{1}$ and the natural death rate $\nu$.

Treatment of an alcohol addictive person is a complex and multifaceted process that often requires a combination of medical, psychological, and social support. Keeping the importance of treatment interventions, in this study, we extend the model^23^ by adding a treated class for the heavy drinkers under the assumption that some of the heavy drinkers become quitter/recovered passing through proper medications. Common medications include naltrexone, acamprosate, and disulfiram^2^. Additionally, unlike the previous work, we assume that the heavy drinkers who exhibit severe alcohol addiction can potentially pass on the addiction to individuals in the class of potential drinkers $\mathcal {P}(t)$. The class of treated individuals formed as a result of treating heavy drinkers at rate $\phi$ and are reducing at the rate $\delta _{2}$ (death rate of treated individuals), $\gamma _{1}$ (the rate at which individuals recover and quit drinking) and $\nu$ of natural mortality rate.

The individuals in violence drinkers class $\mathcal {V}(t)$ engage in lengthy and excessive alcohol consumption and carry out numerous violent activities. The violent heavy drinker’s population class is enhanced by the rate $\theta _{1}$ and reduced at a rate $\gamma _{2}$ (a rate of $\mathcal {V}(t)$ who become healthier and become quitters), and $\delta _{3}$(the alcohol-induced in $\mathcal {V}(t)$ class). Moreover, this class is declined due to natural mortality at a rate $\nu .$

Heavy drinkers who caused road accidents join the class $\mathcal {A}(t)$ by the rate $\theta _{2}$. Moreover, this class is decreased by the quitting rates $\gamma _{3}$, the death rate $\delta _{4}$ induced by $\mathcal {A}(t)$ and the natural motility rate $\nu .$ The recovered or quitter drinkers refer to those who have either temporarily or permanently stopped drinking. This class is increased by the recovery of individuals in various groups at the rates $\gamma _{1},\gamma _{2},\gamma _{3}$ and $\theta _{3}$. The recovered or quitter individuals are decreased due to natural death at a rate $\nu$.

**Figure 1:**
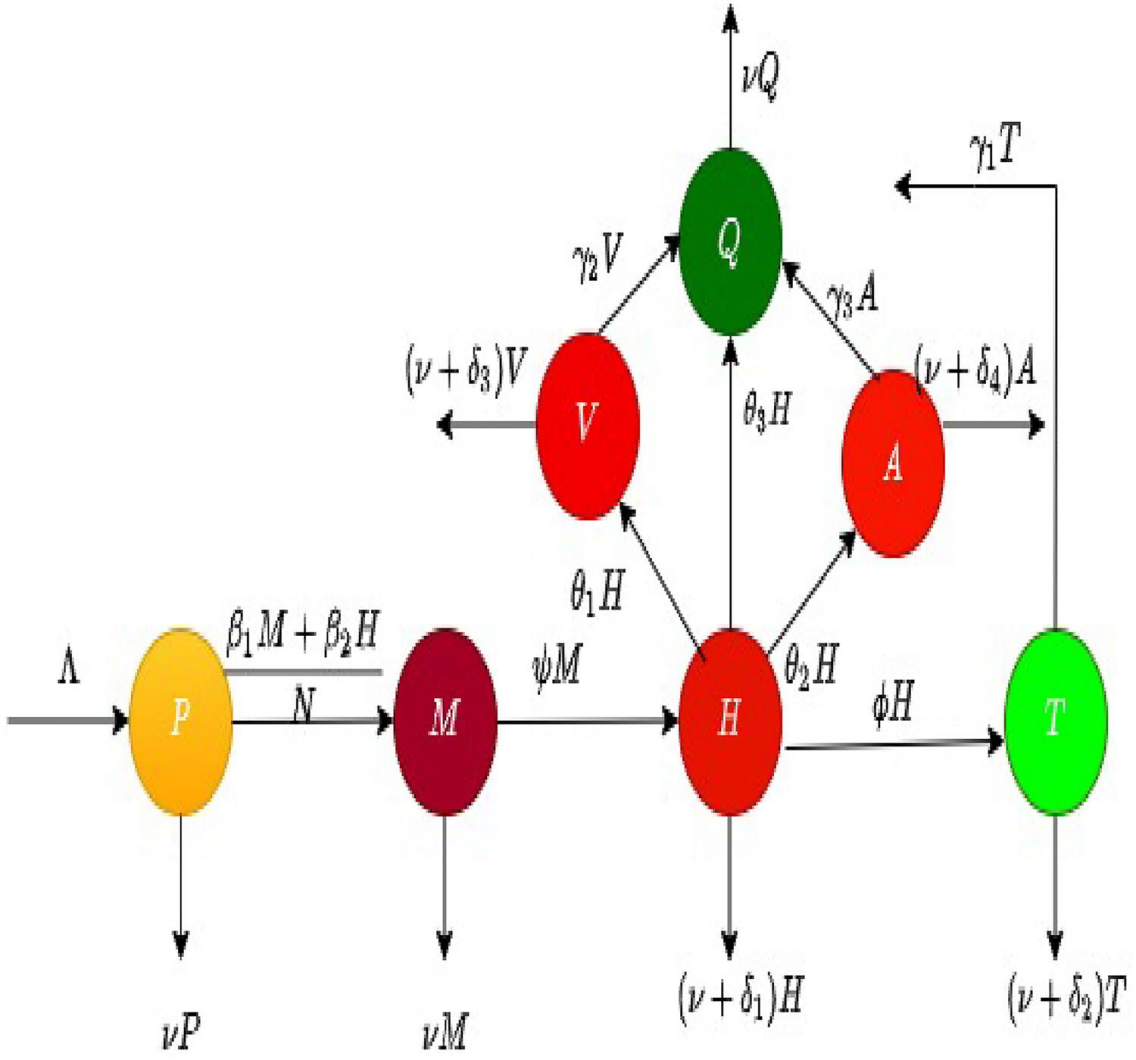
Flow diagram of the proposed alcohol model.

The system of differential equations listed below demonstrates the model’s behavior and the transmission among different compartments is given in Fig. 1.

4 $$\begin{aligned} \frac{d\mathcal {P}}{dt}&= \Lambda - (\lambda +\nu ) \mathcal {P}, \nonumber \\ \frac{d\mathcal {M}}{dt}&= \lambda \mathcal {P} - \left( {\psi + \nu } \right) \mathcal {M}, \nonumber \\ \frac{d\mathcal {H}}{dt}&= \psi \mathcal {M} - \left( {\nu + \theta _1 + \theta _2 + \theta _3 + \delta _1 + \phi } \right) \mathcal {H}, \nonumber \\ \frac{d\mathcal {T}}{dt}&= \phi \mathcal {H} - \left( {\nu + \delta _2 + \gamma _1 } \right) \mathcal {T}, \nonumber \\ \frac{d\mathcal {V}}{dt}&= \theta _1 \mathcal {H} - \left( {\nu + \delta _3 + \gamma _2 } \right) \mathcal {V}, \nonumber \\ \frac{d\mathcal {A}}{dt}&= \theta _2 \mathcal {H}- \left( {\nu + \delta _4 + \gamma _3 } \right) \mathcal {A}, \nonumber \\ \frac{d\mathcal {Q}}{dt}&= \theta _3 \mathcal {H} + \gamma _1 \mathcal {T} + \gamma _2 \mathcal {V} + \gamma _3 \mathcal {A} - \nu \mathcal {Q}, \end{aligned}$$

subject to initial conditions (ICs) $\mathcal {P}\left( 0 \right) = \mathcal {P}_0 ,\,\mathcal {M}\left( 0 \right) = \mathcal {M}_0 ,\,\,\mathcal {H}\left( 0 \right) = \mathcal {H}_0 ,\,\mathcal {T}\left( 0 \right) = \mathcal {T}_0 ,\,\mathcal {V}\left( 0 \right) = \mathcal {V}_0 ,\,\,\mathcal {A}\left( 0 \right) = \mathcal {A}_0 ,\,\mathcal {Q}\left( 0 \right) = \mathcal {Q}_0$, and $\lambda = \frac{{\beta _1 \mathcal {M} + \beta _2 \mathcal {H}}}{\mathcal {N}}.$

## Fractional extension of the alcohol addiction model

In recent years, mathematical modeling using fractional differential equations has been recognized as an effective technique. Based on various features (as mentioned in the introduction), such differential equations are widely used in engineering and sciences such as mechanics, physics, chemistry, economics, and computational biology. The major reason is that fractional differential operators are a valuable tool to observe the influence of memory effects, which exists in most biological systems^13,16^. We reformulate the classical integer order model (4) using the system of fractional differential equations in the Caputo sense. Thus, in the system a time correlation function or memory kernel appears, providing a more accurate representation of the dynamics of such problem^15,16^. The system (4) in the fractional case can be reformulated as follows:

5 $$\begin{aligned} ^{C}D_{t}^{\vartheta }\mathcal {P}(t)&= \Lambda - (\lambda +\nu ) \mathcal {P}, \nonumber \\ ^{C}D_{t}^{\vartheta }\mathcal {M}(t)&= \lambda \mathcal {P} - \left( {\psi + \nu } \right) \mathcal {M}, \nonumber \\ ^{C}D_{t}^{\vartheta }\mathcal {H}(t)&= \psi \mathcal {M} - \left( {\nu + \theta _1 + \theta _2 + \theta _3 + \delta _1 + \phi } \right) \mathcal {H}, \nonumber \\ ^{C}D_{t}^{\vartheta }\mathcal {T}(t)&= \phi \mathcal {H} - \left( {\nu + \delta _2 + \gamma _1 } \right) \mathcal {T}, \nonumber \\ ^{C}D_{t}^{\vartheta }\mathcal {V}(t)&= \theta _1 \mathcal {H} - \left( {\nu + \delta _3 + \gamma _2 } \right) \mathcal {V}, \nonumber \\ ^{C}D_{t}^{\vartheta }\mathcal {A}(t)&= \theta _2 \mathcal {H}- \left( {\nu + \delta _4 + \gamma _3 } \right) \mathcal {A}, \nonumber \\ ^{C}D_{t}^{\vartheta }\mathcal {Q}(t)&= \theta _3 \mathcal {H} + \gamma _1 \mathcal {T} + \gamma _2 \mathcal {V} + \gamma _3 \mathcal {A} - \nu \mathcal {Q}, \end{aligned}$$

where $^{C}D_{t}^{\vartheta }$ is the Caputo derivative having order $\vartheta$.

### Basic analysis

A detailed analysis of the aforementioned alcohol addiction model (5) is provided in this part.

#### Positively invariant region

Through some mathematical results, we can easily demonstrate that the fractional model in Caputo sense has $\frac{\Lambda }{\nu }$ as an upper bound of $\mathcal {N}(t)$ i.e., $\mathcal {N}(t)\le \frac{\Lambda }{\nu },$ if $\mathcal {N}(0)\le \frac{\Lambda }{\nu }.$ Thus, the feasible region is structured as follows:

$$\begin{aligned} \Omega = \left\{ \begin{array}{l} \left( {\mathcal {P}\left( t \right) ,\mathcal {M}\left( t \right) ,\mathcal {H}\left( t \right) ,\mathcal {T}\left( t \right) ,\mathcal {V}\left( t \right) ,\mathcal {A}\left( t \right) ,\mathcal {Q}\left( t \right) } \right) \\ :\,\mathcal {P}\left( t \right) +\mathcal {M}\left( t \right) + \mathcal {H}\left( t \right) + \mathcal {T}\left( t \right) +\mathcal {A}\left( t \right) + \mathcal {V}\left( t \right) + \mathcal {Q}\left( t \right) \le \frac{\Lambda }{\nu } \\ \end{array} \right\} . \end{aligned}$$

The positively invariantness of region $\Omega$ can be shown using Laplace transom.

#### Basic reproductive number and equilibrium states

The alcohol model (5) have two equilibria namely the alcohol-free equilibrium (AFE) $\mathcal {D}^{0}$ and the endemic equilibrium (EE). The AFE can be given as:

$$\begin{aligned} \mathcal {D}^0 = \left( {\mathcal {P},\mathcal {M},\mathcal {H},\mathcal {T},\mathcal {V},\mathcal {A},\mathcal {Q}} \right) = \left( {\Lambda /\nu ,0,0,0,0,0,0} \right) . \end{aligned}$$

Utilizing the well-established method from^35^, we compute the threshold number $\mathcal {R}_{0}$ as follows,

$$\begin{aligned} \mathcal {R}_{0} = \frac{{q_2 \beta _1 + \psi \beta _2 }}{{q_1 q_2 }}. \end{aligned}$$

#### Endemic equilibrium point

The EE, represented by $\mathcal {E^{*}}=(\mathcal {P}^{*},\mathcal {M}^{*},\mathcal {H}^{*},\mathcal {T}^{*},\mathcal {V}^{*},\mathcal {A}^{*},\mathcal {Q}^{*}),$ and given by

6 $$\begin{aligned} \mathcal {P}^*&= \frac{\Lambda }{{\left( {\lambda ^* + \nu } \right) }}, \nonumber \\ \mathcal {M}^*&= \frac{{\Lambda \lambda ^* }}{{\left( {\lambda ^* + \nu } \right) q_1 }} = \frac{{\lambda ^* }}{{q_1 }}\mathcal {P}^* = c_1 \lambda ^* \mathcal {P}^* , \nonumber \\ \mathcal {H}^*&= \frac{{\Lambda \psi \lambda ^* }}{{\left( {\lambda ^* + \nu } \right) q_1 q_2 }} = \frac{{\psi \lambda ^* }}{{q_1 q_2 }}\mathcal {P}^* = c_2 \lambda ^* \mathcal {P}^* , \nonumber \\ \mathcal {T}^*&= \frac{{\Lambda \psi \phi \lambda ^* }}{{\left( {\lambda ^* + \nu } \right) q_1 q_2 q_3 }} = \frac{{\psi \phi \lambda ^* }}{{q_1 q_2 q_3 }}\mathcal {P}^* = c_3 \lambda ^* \mathcal {P}^* , \nonumber \\ \mathcal {V}^*&= \frac{{\Lambda \psi \theta _1 \lambda ^* }}{{\left( {\lambda ^* + \nu } \right) q_1 q_2 q_4 }} = \frac{{\psi \theta _1 \lambda ^* }}{{q_1 q_2 q_4 }}\mathcal {P}^* = c_4 \lambda ^*\mathcal {P}^* , \nonumber \\ \mathcal {A}^*&= \frac{{\Lambda \psi \theta _2 \lambda ^* }}{{\left( {\lambda ^* + \nu } \right) q_1 q_2 q_5 }} = \frac{{\psi \theta _2 \lambda ^* }}{{q_1 q_2 q_5 }}\mathcal {P}^* = c_5 \lambda ^* \mathcal {P}^* , \nonumber \\ \mathcal {Q}^*&= \frac{1}{\nu }\left( {c_2 \theta _3 + c_3\gamma_1 + c_4 \gamma_2+ c_5\gamma_3 } \right) \lambda ^* \mathcal {P}^* = \frac{{c_6 \lambda ^*\mathcal {P}^* }}{\nu }. \end{aligned}$$

Force of infection at endemic point is given below

7 $$\begin{aligned} \lambda ^* = \frac{{\beta _1 \mathcal {M}^* + \beta _2 \mathcal {H}^* }}{{\mathcal {N}^* }}. \end{aligned}$$

By putting (6) in (7) we have

8 $$\begin{aligned} \begin{array}{l} \lambda ^* = \frac{{\mathcal {R}_0 - 1}}{{c_7 }}> 0,\,\,\,\mathcal {R}_0 > 1, \end{array} \end{aligned}$$

where,

$$\begin{aligned} c_7&= \left( {c_1 + c_2 + c_3 + c_4 + c_5 + \frac{{c_6 }}{\nu }} \right) , q_1 = \left( {\psi \mathrm{{ + }}\nu } \right) ,\,\,q_2 = \left( {\nu + \theta _1 + \theta _2 + \theta _3 + \delta _1 + \phi } \right) ,\\ q_3&= \left( {\nu + \delta _2 + \gamma _1 } \right) , q_4 = \left( {\nu + \delta _3 + \gamma _2 } \right) ,\,q_5 = \left( {\nu + \delta _4 + \gamma _3 } \right) . \end{aligned}$$

Thus, a unique EE exists when $\mathcal {R}_{0}$ is greater than 1.

### Uniqueness and existence of the fractional model

We will investigate the uniqueness and existence of the suggested alcohol model in the Caputo sense. The model can be restructured in the subsequent problem

9 $$\begin{aligned} \left\{ \begin{array}{l} ^{C} D_t^\vartheta u\left( t \right) = \mathcal {F}\left( {t,u(t)} \right) , \\ \\ u\left( 0 \right) = u_0 ,\,\,\,0< t< T < \infty , \\ \end{array} \right. \end{aligned}$$

such that $u(t)=(\mathcal {P},\mathcal {M},\mathcal {H},\mathcal {T},\mathcal {V},\mathcal {A},\mathcal {Q})$, presents the subclasses and $\mathcal {F}$ is given below

10 $$\begin{aligned} \mathcal {F}\left( {t,u\left( t \right) } \right) = \left( {\begin{array}{c} {\mathcal {F}_1 \left( {t,\mathcal {P}\left( t \right) } \right) } \\ {\mathcal {F}_2 \left( {t,\mathcal {M}\left( t \right) } \right) } \\ {\mathcal {F}_3 \left( {t,\mathcal {H}\left( t \right) } \right) } \\ {\mathcal {F}_4 \left( {t,\mathcal {T}\left( t \right) } \right) } \\ {\mathcal {F}_5 \left( {t,\mathcal {V}\left( t \right) } \right) } \\ {\mathcal {F}_6 \left( {t,\mathcal {A}\left( t \right) } \right) } \\ {\mathcal {F}_7 \left( {t,\mathcal {Q}\left( t \right) } \right) } \\ \end{array}} \right) = \left( {\begin{array}{c} {\Lambda -(\lambda +\nu ) \mathcal {P}} \\ {\lambda \mathcal {P}- \left( {\psi + \nu } \right) \mathcal {M}} \\ {\psi \mathcal {M} - \left( {\nu + \theta _1 + \theta _2 + \theta _3 + \delta _1 + \phi } \right) \mathcal {H}} \\ {\phi \mathcal {H} - \left( {\nu + \delta _2 + \gamma _1 } \right) \mathcal {T}} \\ {\theta _1 \mathcal {H} - \left( {\nu + \delta _3 + \gamma _2 } \right) \mathcal {V}} \\ {\theta _2 \mathcal {H} - \left( {\nu + \delta _4 + \gamma _3 } \right) \mathcal {A}} \\ {\theta _3 \mathcal {H} + \gamma _1 \mathcal {T} + \gamma _2 \mathcal {V} + \gamma _3 \mathcal {A} - \nu \mathcal {Q}} \\ \end{array}} \right) . \end{aligned}$$

By using the integral operator we obtained

$$\begin{aligned} u(t) = u_0+I_t^\vartheta (\mathcal {F}({t,u(t)})). \end{aligned}$$

After transforming the initial valued problem (IVP) (9) via the Picard iteration, it can be expressed in the following form

11 $$\begin{aligned} u\left( t \right) = u_0 + \frac{1}{{\Gamma \left( \vartheta \right) }}\int \limits _0^t {\left( {\left( {t - r} \right) ^{\vartheta - 1} \mathcal {F}\left( {r,u\left( r\right) } \right) } \right) dr.} \end{aligned}$$

#### Lemma 1

The vector function $\mathcal {F}(t,u(t))$ defined in Eq. (9) satisfies the Lipschitz condition with respect to *u* on the set $\left[ 0, T\right] \times \mathbb {R}_+^7$, and this condition holds with the following Lipschitz constant

$$\begin{aligned} \eta = \max \left( \begin{array}{l} \left( {\beta _1^* + \beta _2^* + \nu } \right) ,\left( {\psi + \nu } \right) ,\left( {\nu + \theta _1 + \theta _2 + \theta _3 + \delta _1 + \phi } \right) , \\ \left( {\nu + \delta _2 + \gamma _1 } \right) ,\left( {\nu + \delta _3 + \gamma _2 } \right) ,\left( {\nu + \delta _4 + \gamma _3 } \right) ,\nu \end{array} \right) . \end{aligned}$$

#### Proof

$$\begin{aligned} \left\| {\mathcal {F}_1 \left( {t,\mathcal {P}} \right) - \mathcal {F}_1 \left( {t,\mathcal {P}_1 } \right) } \right\|&= \left\| {\left( {\Lambda - \frac{{\left( {\beta _1 \mathcal {M} + \beta _2 \mathcal {H}} \right) \mathcal {P}}}{\mathcal {N}} - \nu \mathcal {P}} \right) - \left( {\Lambda - \frac{{\left( {\beta _1 \mathcal {M} + \beta _2 \mathcal {H}} \right) \mathcal {P}_1 }}{\mathcal {N}} - \nu \mathcal {P}_1 } \right) } \right\| \\&= \left\| { - \left( {\left( {\frac{{\left( {\beta _1 \mathcal {M} + \beta _2 \mathcal {H}} \right) }}{\mathcal {N}}} \right) \left( {\mathcal {P} - \mathcal {P}_1 } \right) + \nu \left( {\mathcal {P} - \mathcal {P}_1 } \right) } \right) } \right\| \\\\&\le \left( {\beta _1^* + \beta _2^* + \nu } \right) \left\| {\mathcal {P} - \mathcal {P}_1 } \right\| . \end{aligned}$$

Similarly,

$$\begin{aligned} \left\| {\mathcal {F}_2 \left( {t,\mathcal {M}} \right) -\mathcal {F}_2 \left( {t,\mathcal {M}_1 } \right) } \right\|&\le \left( {\psi + \nu } \right) \left\| {\mathcal {M} - \mathcal {M}_1 } \right\| , \\ \left\| {\mathcal {F}_3 \left( {t,\mathcal {H}} \right) - \mathcal {F}_3 \left( {t,\mathcal {H}_1 } \right) } \right\|&\le \left( {\nu + \theta _1 + \theta _2 + \theta _3 + \delta _1 + \phi } \right) \left\| {\mathcal {H} - \mathcal {H}_1 } \right\| , \\ \left\| {\mathcal {F}_4 \left( {t,\mathcal {T}} \right) - \mathcal {F}_4 \left( {t,\mathcal {T}_1 } \right) } \right\|&\le \left( {\nu + \delta _2 + \gamma _1 } \right) \left\| {\mathcal {T} - \mathcal {T}_1 } \right\| , \\ \left\| {\mathcal {F}_5 \left( {t,V} \right) - \mathcal {F}_5 \left( {t,\mathcal {V}_1 } \right) } \right\|&\le \left( {\nu + \delta _3 + \gamma _2 } \right) \left\| {\mathcal {V} -\mathcal {V}_1 } \right\| , \\ \left\| {\mathcal {F}_6 \left( {t,\mathcal {A}} \right) -\mathcal {F}_6 \left( {t,\mathcal {A}_1 } \right) } \right\|&\le \left( {\nu + \delta _4 + \gamma _3 } \right) \left\| {\mathcal {A} - \mathcal {A}_1 } \right\| , \\ \left\| {\mathcal {F}_7 \left( {t,\mathcal {Q}} \right) - \mathcal {F}_7 \left( {t,\mathcal {Q}_1 } \right) } \right\|&\le \nu \left\| {\mathcal {Q} - \mathcal {Q}_1 } \right\| . \end{aligned}$$

Incorporating all equations, we have

12 $$\begin{aligned} \left\| {F\left( {t,u_1 \left( t \right) } \right) - F\left( {t,u_2 \left( t \right) } \right) } \right\| \le \eta \left\| {u_1 - u_2 } \right\| , \end{aligned}$$

where $\eta$ is defined above. $\square$

#### Lemma 2

The IVP (5) has a unique solution $u(t)\in C(J)$, if the conditions in (12) holds.

#### Proof

To attain the desired result, solution of the (5) is considered as $u(t)=\mathcal {W}(t,u(t)),$ where $\mathcal {W}$ such that $\mathcal {W}:C(J,\mathbb {R}^{7}_{+})\rightarrow C(J,\mathbb {R}^{7}_{+})$, defines the Picard operator given as:

$$\begin{aligned} \mathcal {W}\left( {u\left( t \right) } \right) = u_0 + \frac{1}{{\Gamma \left( \vartheta \right) }}\int \limits _0^t {\left( {t - \varrho } \right) ^{\vartheta - 1} \mathcal {F}\left( {\varrho ,u(\varrho )} \right) d\varrho .} \end{aligned}$$

Moreover, it gives

$$\begin{aligned} \left\| {\mathcal {W}\left( {u_1 \left( t \right) } \right) - \mathcal {W}\left( {u_2 \left( t \right) } \right) } \right\|&= \left\| {\frac{1}{{\Gamma \left( \vartheta \right) }}\int \limits _0^t {\left( {t - \varrho } \right) ^{\vartheta - 1} \left[ {\mathcal {F}\left( {\varrho ,u_1 \left( \varrho \right) } \right) -\mathcal {F}\left( {\varrho ,u_2 \left( \varrho \right) } \right) } \right] d\varrho } } \right\| \\&\le \frac{1}{{\Gamma \left( \vartheta \right) }}\int \limits _0^t {\left( {t - \varrho } \right) ^{\vartheta - 1} } \left\| {\mathcal {F}\left( {\varrho ,u_1 \left( \varrho \right) } \right) - \mathcal {F}\left( {\varrho ,u_2 \left( \varrho \right) } \right) } \right\| d\varrho \\&\le \frac{\eta }{{\Gamma \left( \vartheta \right) }}\int \limits _0^t {\left( {t -\varrho } \right) ^{\vartheta - 1} } \left\| {u_1 - u_2 } \right\| d\varrho \\&\le \frac{\eta }{{\Gamma \left( {\vartheta + 1} \right) }}\mathcal {W}. \end{aligned}$$

If $\frac{\eta \mathcal {W}}{\Gamma (\vartheta +1)}<1,$ then $\mathcal {W}$ gives a contraction and thereby ensuring that there exits unique solution of the problem. $\square$

### Stability analysis

Mathematical stability theory explores the resilience of differential equation solutions, including both fractional and non-fractional forms, and the robustness of dynamical system trajectories when faced with minor changes to their initial states. Ulam–Hyers (UH) and Ulam–Hyers–Rassias (UHR) stability analysis is one of the widely used techniques for evaluating stability in the context of fractional derivatives. UH stability was first discussed in^36^, but in a later publication by Rassias^37^, it was given a more comprehensive sense. When finding precise solutions is difficult, UH and UHR stability offers useful tools for controlling the behavior of a proposed model. Following the UH and UHR stability criteria, we will investigate analogous ideas from^38–40^ while looking at the stable solutions of the Caputo fractional alcohol addiction behavior model (5).

#### Definition 5

The alcohol addiction model in Caputo case (5) is UH stable if there exist $$0<D_{w_{i}}\in \mathbb {R}, i=1,...,7$$ such that $$\forall \delta _{i}>0$$ and $$\forall$$ $$(\mathcal {P}^{***},\mathcal {M}^{***},\mathcal {H}^{***},\mathcal {T}^{***},\mathcal {V}^{***},\mathcal {A}^{***},\mathcal {Q}^{***})\in \mathbb {Y}$$ satisfying

13 $$\begin{aligned} \left\{ \begin{array}{l} \left| {^C D_{t}^\vartheta \mathcal {P}^{***} \left( t \right) - \kappa _1 \left( {\mathcal {P}^{***} ,\mathcal {M}^{***} ,\mathcal {H}^{***} ,\mathcal {T}^{***} ,\mathcal {V}^{***},\mathcal {A}^{***} ,\mathcal {Q}^{***} } \right) (t)} \right|< \delta _1 , \\ \\ \left| {^C D_{t}^\vartheta \mathcal {M}^{***} \left( t \right) - \kappa _2 \left( {\mathcal {P}^{***} ,\mathcal {M}^{***} ,\mathcal {H}^{***} ,\mathcal {T}^{***} ,\mathcal {V}^{***},\mathcal {A}^{***} ,\mathcal {Q}^{***} } \right) (t)} \right|< \delta _2 , \\ \\ \left| {^C D_{t}^\vartheta \mathcal {H}^{***} \left( t \right) - \kappa _3 \left( {\mathcal {P}^{***} ,\mathcal {M}^{***} ,\mathcal {H}^{***} ,\mathcal {T}^{***} ,\mathcal {V}^{***},\mathcal {A}^{***} ,\mathcal {Q}^{***} } \right) (t)} \right|< \delta _3 ,\\ \\ \left| {^C D_{t}^\vartheta \mathcal {T}^{***} \left( t \right) - \kappa _4 \left( {\mathcal {P}^{***} ,\mathcal {M}^{***} ,\mathcal {H}^{***} ,\mathcal {T}^{***} ,\mathcal {V}^{***},\mathcal {A}^{***} ,\mathcal {Q}^{***} } \right) (t)} \right|< \delta _4 ,\\ \\ \left| {^C D_{t}^\vartheta \mathcal {V}^{***} \left( t \right) - \kappa _5 \left( {\mathcal {P}^{***} ,\mathcal {M}^{***} ,\mathcal {H}^{***} ,\mathcal {T}^{***} ,\mathcal {V}^{***},\mathcal {A}^{***} ,\mathcal {Q}^{***} } \right) (t)} \right|< \delta _5 ,\\ \\ \left| {^C D_{t}^\vartheta \mathcal {A}^{***} \left( t \right) - \kappa _6 \left( {\mathcal {P}^{***} ,\mathcal {M}^{***} ,\mathcal {H}^{***} ,\mathcal {T}^{***} ,\mathcal {V}^{***},\mathcal {A}^{***} ,\mathcal {Q}^{***} } \right) (t)} \right|< \delta _6 ,\\ \\ \left| {^C D_{t}^\vartheta \mathcal {Q}^{***} \left( t \right) - \kappa _7 \left( {\mathcal {P}^{***} ,\mathcal {M}^{***} ,\mathcal {H}^{***} ,\mathcal {T}^{***} ,\mathcal {V}^{***},\mathcal {A}^{***} ,\mathcal {Q}^{***} } \right) (t)} \right| < \delta _7 , \end{array} \right. \end{aligned}$$

$\exists \,\ (\mathcal {P},\mathcal {M},\mathcal {H},\mathcal {T},\mathcal {V},\mathcal {A},\mathcal {Q})\in \mathbb {Y}$ satisfying the fractional Caputo model (5) with

14 $$\begin{aligned} \left\{ \begin{array}{l} \left| {\mathcal {P}^{***} - \mathcal {P}} \right|< D_{w_1 } \delta _1 , \\ \\ \left| {\mathcal {M}^{***} - \mathcal {M}} \right|< D_{w_2 } \delta _2 , \\ \\ \left| {\mathcal {H}^{***} - \mathcal {H}} \right|< D_{w_3 } \delta _3 , \\ \\ \left| {\mathcal {T}^{***} - \mathcal {T}} \right|< D_{w_4 } \delta _4 , \\ \\ \left| {\mathcal {V}^{***} - \mathcal {V}} \right|< D_{w_5 } \delta _5 , \\ \\ \left| {\mathcal {A}^{***} - \mathcal {A}} \right|< D_{w_6 } \delta _6 , \\ \\ \left| {\mathcal {Q}^{***} - \mathcal {Q}} \right| < D_{w_7 } \delta _7 . \\ \end{array} \right. \end{aligned}$$

#### Remark 1

$$\left( {\mathcal {P}^{***} ,\mathcal {M}^{***} ,\mathcal {H}^{***} ,\mathcal {T}^{***} ,\mathcal {V}^{***} ,\mathcal {A}^{***} ,\mathcal {Q}^{***} } \right) \in \mathbb {Y}$$ be the solution of (13) iff for $$i=1,\cdots ,7$$ there exists $$p_i \in C\left( {\left[ {0,T} \right] ,\mathbb {R}} \right)$$ (based on $$\left( {\mathcal {P}^{***} ,\mathcal {M}^{***} ,\mathcal {H}^{***} ,\mathcal {T}^{***} ,\mathcal {V}^{***} ,\mathcal {A}^{***} ,\mathcal {Q}^{***} } \right)$$ respectively) such that $$\forall t \in T,$$ we have

- $$\left| {p_i \left( t \right) } \right| < \delta _i,\,\left( {i = 1,\cdots ,7} \right)$$ and
- 15 $$\begin{aligned} \left\{ \begin{array}{l} ^C D_{t}^\vartheta \mathcal {P}^{***} \left( t \right) = \kappa _1 \left( {\mathcal {P}^{***} ,\mathcal {M}^{***} ,\mathcal {H}^{***} ,\mathcal {T}^{***} ,\mathcal {V}^{***},\mathcal {A}^{***} ,\mathcal {Q}^{***} } \right) (t) + p_1 \left( t \right) , \\ \\ ^C D_{t}^\vartheta M^{***} \left( t \right) = \kappa _2 \left( {\mathcal {P}^{***} ,\mathcal {M}^{***} ,\mathcal {H}^{***} ,\mathcal {T}^{***} ,\mathcal {V}^{***},\mathcal {A}^{***} ,\mathcal {Q}^{***} } \right) (t) + p_2 \left( t \right) , \\ \\ ^C D_{t}^\vartheta \mathcal {H}^{***} \left( t \right) = \kappa _3 \left( {\mathcal {P}^{***} ,\mathcal {M}^{***} ,\mathcal {H}^{***} ,\mathcal {T}^{***} ,\mathcal {V}^{***},\mathcal {A}^{***} ,\mathcal {Q}^{***} } \right) (t) + p_3 \left( t \right) , \\ \\ ^C D_{t}^\vartheta \mathcal {T}^{***} \left( t \right) = \kappa _4 \left( {\mathcal {P}^{***} ,\mathcal {M}^{***} ,\mathcal {H}^{***} ,\mathcal {T}^{***} ,\mathcal {V}^{***},\mathcal {A}^{***} ,\mathcal {Q}^{***} } \right) (t) + p_4 \left( t \right) , \\ \\ ^C D_{t}^\vartheta \mathcal {V}^{***} \left( t \right) = \kappa _5 \left( {\mathcal {P}^{***} ,\mathcal {M}^{***} ,\mathcal {H}^{***} ,\mathcal {T}^{***} ,\mathcal {V}^{***},\mathcal {A}^{***} ,\mathcal {Q}^{***} } \right) (t) + p_5 \left( t \right) , \\ \\ ^C D_{t}^\vartheta \mathcal {A}^{***} \left( t \right) = \kappa _6 \left( {\mathcal {P}^{***} ,\mathcal {M}^{***} ,\mathcal {H}^{***} ,\mathcal {T}^{***} ,\mathcal {V}^{***},\mathcal {A}^{***} ,\mathcal {Q}^{***} } \right) (t) + p_6 \left( t \right) , \\ \\ ^C D_{t}^\vartheta \mathcal {Q}^{***} \left( t \right) = \kappa _7 \left( {\mathcal {P}^{***} ,\mathcal {M}^{***} ,\mathcal {H}^{***} ,\mathcal {T}^{***} ,\mathcal {V}^{***},\mathcal {A}^{***} ,\mathcal {Q}^{***} } \right) (t) + p_7 \left( t \right) . \end{array} \right. \end{aligned}$$

#### Definition 6

The fractional Caputo Alcohol addiction behavior model (5) is UHR stable for the functions $$\chi _i,i=1,\cdots ,7$$ whenever there exists $$0<D_{w_{i}},\chi _i\in \mathbb {R},i=1,\cdots ,7$$ such that $$\forall \delta _{i}>0$$ and $$\forall (\mathcal {P}^{***},\mathcal {M}^{***},\mathcal {H}^{***},\mathcal {T}^{***},\mathcal {V}^{***},\mathcal {A}^{***},\mathcal {Q}^{***})\in \mathbb {Y}$$ satisfying

16 $$\begin{aligned} \left\{ \begin{array}{l} \left| {^C D_{t}^\vartheta \mathcal {P}^{***} \left( t \right) - \kappa _1\left( {\mathcal {P}^{***} ,\mathcal {M}^{***} ,\mathcal {H}^{***} ,\mathcal {T}^{***} ,\mathcal {V}^{***},\mathcal {A}^{***} ,\mathcal {Q}^{***} } \right) (t)} \right|< \delta _1 \chi _1 \left( t \right) , \\ \\ \left| {^C D_{t}^\vartheta \mathcal {M}^{***} \left( t \right) - \kappa _2 \left( {\mathcal {P}^{***} ,\mathcal {M}^{***} ,\mathcal {H}^{***} ,\mathcal {T}^{***} ,\mathcal {V}^{***},\mathcal {A}^{***} ,\mathcal {Q}^{***} } \right) (t)} \right|< \delta _2 \chi _2 \left( t \right) , \\ \\ \left| {^C D_{t}^\vartheta \mathcal {H}^{***} \left( t \right) - \kappa _3 \left( {\mathcal {P}^{***} ,\mathcal {M}^{***} ,\mathcal {H}^{***} ,\mathcal {T}^{***} ,\mathcal {V}^{***},\mathcal {A}^{***} ,\mathcal {Q}^{***} } \right) (t)} \right|< \delta _3 \chi _3 \left( t \right) , \\ \\ \left| {^C D_{t}^\vartheta \mathcal {T}^{***} \left( t \right) - \kappa _4 \left( {\mathcal {P}^{***} ,\mathcal {M}^{***} ,\mathcal {H}^{***} ,\mathcal {T}^{***} ,\mathcal {V}^{***},\mathcal {A}^{***} ,\mathcal {Q}^{***} } \right) (t)} \right|< \delta _4 \chi _4 \left( t \right) , \\ \\ \left| {^C D_{t}^\vartheta \mathcal {V}^{***} \left( t \right) - \kappa _5 \left( {\mathcal {P}^{***} ,\mathcal {M}^{***} ,\mathcal {H}^{***} ,\mathcal {T}^{***} ,\mathcal {V}^{***},\mathcal {A}^{***} ,\mathcal {Q}^{***} } \right) (t)} \right|< \delta _5 \chi _5 \left( t \right) , \\ \\ \left| {^C D_{t}^\vartheta \mathcal {A}^{***} \left( t \right) - \kappa _6 \left( {\mathcal {P}^{***} ,\mathcal {M}^{***} ,\mathcal {H}^{***} ,\mathcal {T}^{***} ,\mathcal {V}^{***},\mathcal {A}^{***} ,\mathcal {Q}^{***} } \right) (t)} \right|< \delta _6 \chi _6 \left( t \right) , \\ \\ \left| {^C D_{t}^\vartheta \mathcal {Q}^{***} \left( t \right) - \kappa _7 \left( {\mathcal {P}^{***} ,\mathcal {M}^{***} ,\mathcal {H}^{***} ,\mathcal {T}^{***} ,\mathcal {V}^{***},\mathcal {A}^{***} ,\mathcal {Q}^{***} } \right) (t)} \right| < \delta _7 \chi _7 \left( t \right) . \\ \\ \end{array} \right. \end{aligned}$$

There exists $(\mathcal {P},\mathcal {M},\mathcal {H},\mathcal {T},\mathcal {V},\mathcal {A},\mathcal {Q})\in \mathbb {Y}$ fulfilling the fractional model (5) together with

17 $$\begin{aligned} \left\{ \begin{array}{l} \left| {\mathcal {P}^{***} \left( t \right) - \mathcal {P}\left( t \right) } \right| \le D_{w_1 ,\chi _1 } \delta _1 \chi _1 \left( t \right) , \\ \\ \left| {\mathcal {M}^{***} \left( t \right) -\mathcal {M}\left( t \right) } \right| \le D_{w_2 ,\chi _2 } \delta _2 \chi _2 \left( t \right) , \\ \\ \left| {\mathcal {H}^{***} \left( t \right) - \mathcal {H}\left( t \right) } \right| \le D_{w_3 ,\chi _3 } \delta _3 \chi _3 \left( t \right) , \\ \\ \left| {\mathcal {T}^{***} \left( t \right) - \mathcal {T}\left( t \right) } \right| \le D_{w_4 ,\chi _4 } \delta _4 \chi _4 \left( t \right) , \\ \\ \left| {\mathcal {V}^{***} \left( t \right) - \mathcal {V}\left( t \right) } \right| \le D_{w_5 ,\chi _5 } \delta _5 \chi _5 \left( t \right) , \\ \\ \left| {\mathcal {A}^{***} \left( t \right) - \mathcal {A}\left( t \right) } \right| \le D_{w_6 ,\chi _6 } \delta _6 \chi _6 \left( t \right) , \\ \\ \left| {\mathcal {Q}^{***} \left( t \right) - \mathcal {Q}\left( t \right) } \right| \le D_{w_7 ,\chi _7 } \delta _7 \chi _7 \left( t \right) . \\ \end{array} \right. \end{aligned}$$

#### Remark 2

$$(\mathcal {P}^{***},\mathcal {M}^{***},\mathcal {H}^{***},\mathcal {T}^{***},\mathcal {V}^{***},\mathcal {A}^{***},\mathcal {Q}^{***})\in \mathbb {Y}$$ is a solution of (16) iff for $$j=1,\cdots ,7,$$ there exists $$p_j \in C\left( {\left[ {0,T} \right] ,\mathbb {R}} \right) ,$$ based on $$\mathcal {P}^{***},\mathcal {M}^{***},\mathcal {H}^{***},\mathcal {T}^{***},\mathcal {V}^{***},\mathcal {A}^{***},\mathcal {Q}^{***}$$ respectively, such that $$\forall t \in T$$

- $$\left| {p_i \left( t \right) } \right| < \chi _i \left( t \right) \delta _i \,,i = 1,\cdots ,7.$$
- The conditions in (15) holds.

#### Theorem 1

The addiction model (5) is UH stable on [0, *T*] such that $\Upsilon \Theta _{i} < 1,\,\,\,\,\,i \in \left\{ {1,\cdots ,7} \right\} ,$ where $\Theta _{i}$ and $\Upsilon$ are given as

$$\begin{aligned} \begin{array}{l} \Theta _i :\,\,\Theta _1 = \beta _1 \Delta _1 + \beta _2 \Delta _2 + \nu ;\,\,\Theta _2 = q_2 ;\,\Theta _3 = q_3 ;\,\Theta _4 = q_4 ;\,\Theta _5 = q_5;\,\Theta _6 = q_6;\\ \\ \Delta _3 = \frac{{t^\vartheta }}{{\Gamma \left( {\vartheta + 1} \right) }}. \end{array} \end{aligned}$$

If the assumptions (D1) given by $\left\| {\frac{\mathcal {M}}{\mathcal {N}}} \right\| \le \Delta _1 ,\,\,\left\| {\frac{\mathcal {H}}{\mathcal {N}}} \right\| \le \Delta _2$ are true.

#### Proof

Let $$\delta _{1}>0$$ and $$\mathcal {P}^{***}\in \mathbb {Y}$$ such that

$$\begin{aligned} \left| {^C D_t^\vartheta \mathcal {P}^{***} - \kappa _1 \left( {\mathcal {P}^{***},\mathcal {M}^{***} ,\mathcal {H}^{***} ,\mathcal {T}^{***} ,\mathcal {V}^{***} ,\mathcal {A}^{***} ,\mathcal {Q}^{***} } \right) } \right| < \delta _1. \end{aligned}$$

Following remark 1, there exists a function $p_{1}(t)$ such that

$$\begin{aligned} ^C D_t^\vartheta \mathcal {P}^{***} = \kappa _1 \left( {\mathcal {P}^{***} ,\mathcal {M}^{***} ,\mathcal {H}^{***} ,\mathcal {T}^{***} ,\mathcal {V}^{***} ,\mathcal {A}^{***} ,\mathcal {Q}^{***} } \right) + p_1 \left( t \right) , \end{aligned}$$

and $$\left| {p_1 \left( t \right) } \right| \le \delta _1$$. Therefore,

$$\begin{aligned} \mathcal {P}^{***} \left( t \right)&= \mathcal {P}_0 + \frac{1}{{\Gamma \left( \vartheta \right) }}\int \limits _0^t {\left( {t - r} \right) ^{\vartheta - 1} \kappa _1 \left( \begin{array}{l} r,\mathcal {P}^{***} \left( r \right) ,\mathcal {M}^{***} \left( r \right) ,\mathcal {H}^{***} \left( r \right) , \\ \mathcal {T}^{***} \left( r \right) ,V^{***} \left( r \right) ,\mathcal {A}^{***} \left( r \right) ,\mathcal {Q}^{***} \left( r \right) \\ \end{array} \right) dr} \\&\quad + \frac{1}{{\Gamma \left( \vartheta \right) }}\int \limits _0^t {\left( {t - r} \right) ^{\vartheta - 1} p_1 \left( r \right) dr} . \\ \end{aligned}$$

From Lemma (2), we assume $\mathcal {P} \in \mathbb {Y}$ as the unique solution of the system in fractional case, then $\mathcal {P}(t)$ is expressed as below

$$\begin{aligned} \mathcal {P}^{***} \left( t \right) = \mathcal {P}_0 + \frac{1}{{\Gamma \left( \vartheta \right) }}\int \limits _0^t {\left( {t - r} \right) ^{\vartheta - 1} \kappa _1 \left( \begin{array}{l} r,\mathcal {P}^{***} \left( r \right) ,\mathcal {M}^{***} \left( r \right) ,\mathcal {H}^{***} \left( r \right) , \\ \mathcal {T}^{***} \left( r \right) ,\mathcal {V}^{***} \left( r \right) ,\mathcal {A}^{***} \left( r \right) ,\mathcal {Q}^{***} \left( r \right) \\ \end{array} \right) dr}. \end{aligned}$$

Then,

$$\begin{aligned} \left| {\mathcal {P}^{***} \left( t \right) - \mathcal {P}\left( t \right) } \right|&\le \frac{1}{{\Gamma \left( \vartheta \right) }}\int \limits _0^t {\left( {t - r} \right) ^{\vartheta - 1} \left| {p_1 \left( r \right) } \right| dr} + \frac{1}{{\Gamma \left( \vartheta \right) }} \\&\quad \int \limits _0^t {\left( {t - r} \right) ^{\vartheta - 1} \times \left| \begin{array}{l} \kappa _1 \left( r,\mathcal {P}^{***}(r),\mathcal {M}^{***} (r),\mathcal {H}^{***} (r), \mathcal {T}^{***}(r),\mathcal {V}^{***}(r),\mathcal {A}^{***}(r),\mathcal {Q}^{***}(r) \right) \\ - \kappa _1 \left( r,\mathcal {P}(r),\mathcal {M}(r),\mathcal {H}(r),\mathcal {T}(r),\mathcal {V}(r),\mathcal {A}(r),\mathcal {Q}(r)\right) \end{array} \right| dr,} \\&\le \Delta _3 \delta _1 + \Delta _3 \Theta _1 \left\| {\mathcal {P}^{***} - \mathcal {P}} \right\| . \end{aligned}$$

Hence, by applying the supremum norm to both sides of the inequality, we obtain

$$\begin{aligned} \left\| {\mathcal {P}^{***} - \mathcal {P}} \right\| - \Delta _3 \Theta _1 \left\| {\mathcal {P}^{***} - \mathcal {P}} \right\| \le \Delta _3 \delta _1 . \end{aligned}$$

Therefore,

$$\begin{aligned} \left\| {\mathcal {P}^{***} - \mathcal {P}} \right\| \, \le \frac{{\Delta _3 \delta _1 }}{{1 - \Delta _3 \Theta _1 }}. \end{aligned}$$

If $$D_{w_1 } = \frac{{\Delta _3 }}{{1 - \Delta _3 \Theta _1 }},$$ then $$\left\| {\mathcal {P}^{***} - \mathcal {P}} \right\| \le D_{w_1 } \delta _1.$$ In the same way,

$$\begin{aligned}&\left\| {\mathcal {M}^{***} - \mathcal {M}} \right\| \le D_{w_2 } \delta _2 ,\,\,\,\left\| {\mathcal {H}^{***} - \mathcal {H}} \right\| \le D_{w_3 } \delta _3 ,\,\,\,\left\| {\mathcal {T}^{***} - \mathcal {T}} \right\| \le D_{w_4 } \delta _4 ,\, \\&\left\| {\mathcal {V}^{***} - \mathcal {V}} \right\| \le D_{w_5 } \delta _5 ,\,\,\,\,\,\,\left\| {\mathcal {A}^{***} - \mathcal {A}} \right\| \le D_{w_6 } \delta _6 ,\,\,\,\,\left\| {\mathcal {Q}^{***} - \mathcal {Q}} \right\| \le D_{w_7 } \delta _7 , \end{aligned}$$

where

$$\begin{aligned} D_{w_i } = \frac{{\Delta _3 }}{{1 - \Delta _3 \Theta _i }},\,\,i \in \left\{ {2,3,4,5,6,7} \right\} . \end{aligned}$$

Thus, we accomplish the UH stability of the fractional alcohol model. $\square$

#### Theorem 2

Consider the conation *D*1 characterized by the existence of a non-decreasing function $\omega _{i}\in C([0,T],\mathbb {R}^{+})$ for $i\in \{1,2,\cdots ,7\}$ along with the presence of a positive constant $\Xi _{\Omega _i }>0$ such that for all $t\in [0,T]$ we have

18 $$\begin{aligned} ^C I_t^\vartheta \Omega _i \left( t \right) < \Xi _{\Omega _i } \Omega _i \left( t \right) ,\,\,i \in \left\{ {1,\cdots ,7} \right\} . \end{aligned}$$

If the criteria (18) archived then, the UHR stability of the alcohol fractional model (5) is proved.

#### Proof

For each $$\delta _{1}>0$$ and for all $$\mathcal {P}^{***}\in \mathbb {Y}$$ such that

$$\begin{aligned} \left| {^C D_t^\vartheta \mathcal {P}^{***} \left( t \right) - \kappa _1 \left( {t,\mathcal {P}^{***} \left( t \right) ,\mathcal {M}^{***} \left( t \right) ,\mathcal {H}^{***} \left( t \right) ,\mathcal {T}^{***} \left( t \right) ,\mathcal {V}^{***} \left( t \right) ,\mathcal {A}^{***} \left( t \right) ,\mathcal {Q}^{***} \left( t \right) } \right) } \right| < \delta _1 \Omega _1 \left( t \right) , \end{aligned}$$

there exists $p_{1}(t)$ such that

$$\begin{aligned} \begin{array}{l} ^C D_t^\vartheta \mathcal {P}^{***} \left( t \right) = \kappa _1 \left( {t,\mathcal {P}^{***} \left( t \right) ,\mathcal {M}^{***} \left( t \right) ,\mathcal {H}^{***} \left( t \right) ,\mathcal {T}^{***} \left( t \right) ,\mathcal {V}^{***} \left( t \right) ,\mathcal {A}^{***} \left( t \right) ,\mathcal {Q}^{***} \left( t \right) } \right) , \\ \\ and\,\,\,\left| {p_1 \left( t \right) } \right| \le \delta _1 \Omega _1 \left( t \right) . \end{array} \end{aligned}$$

Hence,

$$\begin{aligned} \mathcal {P}^{***} \left( t \right)&= \mathcal {P}_0 + \frac{1}{{\Gamma \left( \vartheta \right) }}\int \limits _0^t \left( {t - r} \right) ^{\vartheta - 1} \kappa _1 \left( {\mathcal {P}^{***} \left( r \right) ,\mathcal {M}^{***} \left( r \right) ,\mathcal {H}^{***} \left( r \right) ,\mathcal {T}^{***} \left( r \right) ,\mathcal {V}^{***} \left( r \right) ,\mathcal {A}^{***} \left( r \right) ,\mathcal {Q}^{***} \left( r \right) } \right) \\&\quad dr+ \frac{1}{{\Gamma \left( \vartheta \right) }}\int \limits _0^t {\left( {t - r} \right) ^{\vartheta - 1} p_1 \left( r \right) dr}. \end{aligned}$$

Based on the information in Lemma (2), the assumption of the existence of unique solution for (5) with $\mathcal {P}\in \mathbb {Y}$ is imposed. Therefore, $\mathcal {P}(t)$ is

$$\begin{aligned} \mathcal {P}^{***} \left( t \right) = \mathcal {P}_0 + \frac{1}{{\Gamma \left( \vartheta \right) }}\int \limits _0^t \left( {t - r} \right) ^{\vartheta - 1} \kappa _1 \left( {\mathcal {P}^{***},\mathcal {M}^{***},\mathcal {H}^{***},\mathcal {T}^{***},\mathcal {V}^{***},\mathcal {A}^{***},\mathcal {Q}^{***} } \right) dr. \end{aligned}$$

We get

$$\begin{aligned} \left| {\mathcal {P}^{***} \left( t \right) - \mathcal {P}\left( t \right) } \right|&\le \frac{1}{{\Gamma \left( \vartheta \right) }}\int \limits _0^t {\left( {t - r} \right) ^{\vartheta - 1} \left| {p_1 \left( r \right) } \right| dr + } \frac{1}{{\Gamma \left( \vartheta \right) }} \\&\quad \int \limits _0^t {\left( {t - r} \right) ^{\vartheta - 1} \times \left| \begin{array}{l} \kappa _1 \left( {r,\mathcal {P}^{***} ,\mathcal {M}^{***} ,\mathcal {H}^{***} ,\mathcal {T}^{***} ,\mathcal {V}^{***} ,\mathcal {A}^{***} ,\mathcal {Q}^{***} } \right) - \\ \kappa _1 \left( {r,\mathcal {P},\mathcal {M},\mathcal {H},\mathcal {T},\mathcal {V},\mathcal {A},\mathcal {Q}} \right) dr \\ \end{array} \right| } \\&\quad \le \frac{{\delta _1 }}{{\Gamma \left( \vartheta \right) }}\int \limits _0^t {\left( {t - r} \right) ^{\vartheta - 1} \Omega _1 \left( r \right) dr + \Delta _3 \Theta _1 \left\| {\mathcal {P}^{***} - \mathcal {P}} \right\| } , \\&\quad \le \delta _1 \Xi _{\Omega _1 } \Omega _1 \left( t \right) + \Delta _3 \Theta _1 \left\| {\mathcal {P}^{***} - \mathcal {P}} \right\| . \end{aligned}$$

Therefore,

$$\begin{aligned} \left\| {\mathcal {P}^{***} - \mathcal {P}} \right\| \le \frac{{\delta _1 \Xi _{\Omega _1 } \Omega _1 \left( t \right) }}{{1 - \Delta _3 \Theta _1 }}. \end{aligned}$$

Further, if

$$\begin{aligned} D_{w_1 ,\Omega _1 } = \frac{{\Xi _{\Omega _1 } }}{{1 - \Delta _3 \Theta _1 }}, \end{aligned}$$

then $$\left\| {\mathcal {P}^{***} - \mathcal {P}} \right\| \le \delta _1 D_{w_1 ,\Omega _1 } \Omega _1 \left( t \right) .$$ In similar way, we have

$$\begin{aligned}&\left\| {\mathcal {M}^{***} - \mathcal {M}} \right\| \le \delta _2 D_{w_2 ,\Omega _2 } \Omega _2 \left( t \right) ,\,\left\| {\mathcal {H}^{***} - \mathcal {H}} \right\| \le \delta _3 D_{w_3 ,\Omega _3 } \Omega _3 \left( t \right) ,\,\left\| {\mathcal {T}^{***} - \mathcal {T}} \right\| \le \delta _4 D_{w_4 ,\Omega _4 } \Omega _4 \left( t \right) , \\&\left\| {\mathcal {V}^{***} -\mathcal {V}} \right\| \le \delta _5 D_{w_5 ,\Omega _5 } \Omega _5 \left( t \right) ,\,\,\,\,\,\,\,\left\| {\mathcal {A}^{***} - \mathcal {A}} \right\| \le \delta _6 D_{w_6 ,\Omega _6 } \Omega _6 \left( t \right) ,\,\,\left\| {\mathcal {Q}^{***} - \mathcal {Q}} \right\| \le \delta _7 D_{w_7 ,\Omega _7 } \Omega _7 \left( t \right) , \end{aligned}$$

where

$$\begin{aligned} D_{w_i ,\Omega _i } = \frac{{\Xi _{\Omega _i } }}{{1 - \Delta _3 \Theta _i }},\,\,\left( {i \in \left\{ {1,2,3,4,5,6,7} \right\} } \right) . \end{aligned}$$

Hence, we conclude that the fractional model (5) is UHR stable. $\square$

## Numerical scheme and simulation discussion

### Numerical scheme

This subsection presents an efficient numerical approach for obtaining an approximate solution to the Caputo alcohol addiction behavior model. To achieve this, the Adams–Bashforth–Moulton scheme is utilized. This technique provides a convergent and stable solution of the proposed fractional model. We rewrite the system (5) as follows:

19 $$\begin{aligned} \left\{ \begin{array}{l} ^c D_t^\vartheta y\left( t \right) = \mathcal {F}\left( {t,y \left( t \right) } \right) ,\,\,0< t < T, \\ y ^{\left( m \right) } \left( 0 \right) = y _0^{\left( m \right) } ,\,\,\,m = 0,1,2,...,\ell ,\,\,\ell = \left[ \vartheta \right] ,\,\,\, \\ \end{array} \right. \end{aligned}$$

where, $y = \left( {\mathcal {P},\mathcal {M},\mathcal {H},\mathcal {V},\mathcal {T},\mathcal {A},\mathcal {Q}} \right) \in \mathbb {R}_ + ^7$ and $\mathcal {F}(t,y(t))$ shows a real-valued and continuous function. Utilizing the Caputo integral, Eq. (19) gives

$$\begin{aligned} y \left( t \right) = \sum \limits _{m = 0}^{v - 1} {y _0^{\left( m \right) } \frac{{t^m }}{{m!}} + \frac{1}{{\Gamma \left( \vartheta \right) }}\int \limits _0^t {\left( {t - x} \right) ^{\vartheta - 1} \mathcal {F}\left( {x,y \left( x \right) } \right) dx.} } \end{aligned}$$

To apply the procedure outlined in^41^, we consider a uniform grid on [0, *T*] with a step size of $h=\frac{T}{N}$, where $N \in \mathbb {N}$. This results in a discrete-time points $t_{u}=uh$ for $u=0,1,2,...,N$. In a consequence, we formulate the iterative scheme for the proposed model (5) as follows:

$$\begin{aligned} \mathcal {P}_{u + 1} \left( t \right)&= \mathcal {P}_0 + \frac{{h^\vartheta }}{{\Gamma \left( {\vartheta + 2} \right) }}\left\{ {\Lambda - \frac{{\left( {\beta _1 \mathcal {M}^m + \beta _2 \mathcal {H}^m } \right) \mathcal {P}^m }}{{\mathcal {N}^m }} - \nu \mathcal {P}^m } \right\} + \frac{{h^\vartheta }}{{\Gamma \left( {\vartheta + 2} \right) }} \\&\quad \sum \limits _{k = 0}^u {\theta _{k,u + 1} \left\{ {\Lambda - \frac{{\left( {\beta _1 \mathcal {M}_k + \beta _2 \mathcal {H}_k } \right) \mathcal {P}_k }}{{\mathcal {N}_k }} - \nu \mathcal {P}_k } \right\} } , \\ \mathcal {M}_{u + 1} \left( t \right)&= \mathcal {M}_0 + \frac{{h^\vartheta }}{{\Gamma \left( {\vartheta + 2} \right) }}\left\{ {\frac{{\left( {\beta _1 \mathcal {M}^m + \beta _2 \mathcal {H}^m } \right) \mathcal {P}^m }}{{\mathcal {N}^m }} - q_1 \mathcal {M}^m } \right\} + \frac{{h^\vartheta }}{{\Gamma \left( {\vartheta + 2} \right) }} \\&\quad \sum \limits _{k = 0}^u {\theta _{k,u + 1} \left\{ {\frac{{\left( {\beta _1 \mathcal {M}_k + \beta _2 \mathcal {H}_k } \right) \mathcal {P}_k }}{{\mathcal {N}_k }} - q_1 \mathcal {M}_k } \right\} } , \\ \mathcal {H}_{u + 1} \left( t \right)&= \mathcal {H}_0 + \frac{{h^\vartheta }}{{\Gamma \left( {\vartheta + 2} \right) }}\left\{ {\psi \mathcal {M}^m - q_2 \mathcal {H}^m } \right\} + \frac{{h^\vartheta }}{{\Gamma \left( {\vartheta + 2} \right) }}\sum \limits _{k = 0}^u {\theta _{k,u + 1} \left\{ {\psi \mathcal {M}_k - q_2 \mathcal {H}_k } \right\} } , \\ \mathcal {T}_{u + 1} \left( t \right)&= \mathcal {T}_0 + \frac{{h^\vartheta }}{{\Gamma \left( {\vartheta + 2} \right) }}\left\{ {\phi \mathcal {H}^m - q_3 \mathcal {T}^m } \right\} + \frac{{h^\vartheta }}{{\Gamma \left( {\vartheta + 2} \right) }}\sum \limits _{k = 0}^u {\theta _{k,u + 1} \left\{ {\phi \mathcal {H}_k - q_3 \mathcal {T}_k } \right\} } , \\ \mathcal {V}_{u + 1} \left( t \right)&= \mathcal {V}_0 + \frac{{h^\vartheta }}{{\Gamma \left( {\vartheta + 2} \right) }}\left\{ {\theta _1 \mathcal {H}^m - q_4 \mathcal {V}^m } \right\} + \frac{{h^\vartheta }}{{\Gamma \left( {\vartheta + 2} \right) }}\sum \limits _{k = 0}^u {\theta _{k,u + 1} \left\{ {\theta _1 \mathcal {H}_k - q_4 \mathcal {V}_k } \right\} } , \\ \mathcal {A}_{u + 1} \left( t \right)&= \mathcal {A}_0 + \frac{{h^\vartheta }}{{\Gamma \left( {\vartheta + 2} \right) }}\left\{ {\theta _2 \mathcal {H}^m - q_5 \mathcal {A}^m } \right\} + \frac{{h^\vartheta }}{{\Gamma \left( {\vartheta + 2} \right) }}\sum \limits _{k = 0}^u {\theta _{k,u + 1} \left\{ {\theta _2 \mathcal {H}_k - q_5 \mathcal {A}_k } \right\} } , \\ \mathcal {Q}_{u + 1} \left( t \right)&= \mathcal {Q}_0 + \frac{{h^\vartheta }}{{\Gamma \left( {\vartheta + 2} \right) }}\left\{ {\theta _3 \mathcal {H}^m + \gamma _1 \mathcal {T}^m + \gamma _2 \mathcal {V}^m + \gamma _3 \mathcal {A}^m - q_4 \mathcal {V}^m } \right\} + \frac{{h^\vartheta }}{{\Gamma \left( {\vartheta + 2} \right) }}\sum \limits _{k = 0}^u {\theta _{k,u + 1} }\\&\quad \left\{ {\theta _3 \mathcal {H}_k + \gamma _1 \mathcal {T}_k + \gamma _2 \mathcal {V}_k + \gamma _3 \mathcal {A}_k - q_4 \mathcal {V}_k } \right\} , \end{aligned}$$

where,

$$\begin{aligned} \mathcal {P}_{u + 1}^m \left( t \right)&=\mathcal {P}_0 + \frac{1}{{\Gamma \left( \vartheta \right) }}\,\sum \limits _{k = 0}^u {b_{k,u + 1} \left\{ {\Lambda - \frac{{\left( {\beta _1 \mathcal {M}_k + \beta _2 \mathcal {H}_k } \right) \mathcal {P}_k }}{{\mathcal {N}_k }} - \nu \mathcal {P}_k } \right\} } , \\ \mathcal {M}_{u + 1}^m \left( t \right)&=\mathcal {M}_0 + \frac{1}{{\Gamma \left( \vartheta \right) }}\,\sum \limits _{k = 0}^u {b_{k,u + 1} \left\{ {\frac{{\left( {\beta _1 \mathcal {M}_k + \beta _2 \mathcal {H}_k } \right) \mathcal {P}_k }}{{\mathcal {N}_k }} - q_1 \mathcal {M}_k } \right\} } , \\ \mathcal {H}_{u + 1}^m \left( t \right)&= \mathcal {H}_0 + \frac{1}{{\Gamma \left( \vartheta \right) }}\sum \limits _{k = 0}^u {b_{k,u + 1} \left\{ {\psi \mathcal {M}_k - q_2 \mathcal {H}_k } \right\} } , \\ \mathcal {T}_{u + 1}^m \left( t \right)&= \mathcal {T}_0 + \frac{1}{{\Gamma \left( \vartheta \right) }}\sum \limits _{k = 0}^u {b_{k,u + 1} \left\{ {\phi \mathcal {H}_k - q_3 \mathcal {T}_k } \right\} } , \\ \mathcal {V}_{u + 1}^m \left( t \right)&= \mathcal {V}_0 + \frac{1}{{\Gamma \left( \vartheta \right) }}\sum \limits _{k = 0}^u {b_{k,u + 1} \left\{ {\theta _1 \mathcal {H}_k - q_4 \mathcal {V}_k } \right\} } , \\ \mathcal {A}_{u + 1}^m \left( t \right)&= \mathcal {A}_0 + \frac{1}{{\Gamma \left( \vartheta \right) }}\sum \limits _{k = 0}^u {b_{k,u + 1} \left\{ {\theta _2 \mathcal {H}_k - q_5 \mathcal {A}_k } \right\} } , \\ \mathcal {Q}_{u + 1}^m \left( t \right)&= \mathcal {Q}_0 + \frac{1}{{\Gamma \left( \vartheta \right) }}\sum \limits _{k = 0}^u {b_{k,u + 1} } \left\{ {\theta _3 \mathcal {H}_k + \gamma _1 \mathcal {T}_k + \gamma _2 \mathcal {V}_k + \gamma _3 \mathcal {A}_k - q_4 \mathcal {V}_k } \right\} . \end{aligned}$$

Furthermore, in the expressions provided above, we have the following:

$$\begin{aligned} \theta _{k,u + 1} = \left\{ \begin{array}{l} u^{\vartheta + 1} - \left( { 1+\ell } \right) \left( {\ell - \vartheta } \right) ,\,\,for \,\,k = 0 \\ \left( {2+\ell - k } \right) ^{\vartheta + 1} + \left( {\ell - k} \right) ^{\vartheta + 1} - 2\left( {\ell + 1- k} \right) ^{\vartheta + 1} ,\,1 \le k \le \ell , \\ 1,\,\,\,k = 1+\ell . \\ \end{array} \right. \end{aligned}$$

and

$$\begin{aligned} b _{k,u + 1} = \frac{{h^\vartheta }}{\vartheta }\left[ {\left( {\ell - k + 1} \right) ^\vartheta + \left( {\ell - k} \right) ^\vartheta } \right] ,\,\,\,\,\,\,0 \le k \le \ell . \end{aligned}$$

**Table 1:** Description of the alcohol model’s parameters.

Parameter	Description	Value	References
$\Lambda$	Recruitment rate of potential individuals	159.5	^23^
$\nu$	Natural death rate of humans	0.1595	^23^
$\beta _1$	Contact rate of potential individuals with moderate individuals	0.4	^23^
$\beta _2$	Contact rate of potential individuals with heavy drinkers	0.00001	Assumed
$\psi$	Proportion of moderate individuals that join heavy drinkers	0.3	^23^
$\phi$	The proportion of drinkers who enter treatment class	$0.70$	^42^
$\theta _{1}$	The rate at which individuals classified as heavy drinkers undergo heavy drinkers class leading to violence	0.03	^23^
$\theta _{2}$	The flow rate from $\mathcal {H}$ to $\mathcal {A}$	0.02	^23^
$\theta _{3}$	The recovery or quitting rate of the heavy drinkers	0.3	^23^
$\gamma _{1}$	The rate at which treated drinkers recover and cease drinking	$\frac{0.0020}{52}$	^42^
$\gamma _{2}$	The recovery or quitting rate of violent heavy drinkers	0.001	^23^
$\gamma _{3}$	The recovery or quitting rate of individuals in $\mathcal {A}$	0.001	^23^
$\delta _{1}$	The mortality rate attributed to heavy drinkers	0.035	^23^
$\delta _{2}$	The induced death rate of treated individuals	$0.30$	^42^
$\delta _{3}$	The mortality rate caused by violence related to alcohol consumption	0.002	^23^
$\delta _{4}$	The motility induced byroad accidents due to heavily drinking	0.001	^23^

### Results and discussion

The alcohol addiction fractional model (5) is simulated in this part using the Adams–Bashforth moulton method with the Caputo operator.

#### Effect of memory index

**Figure 2:**
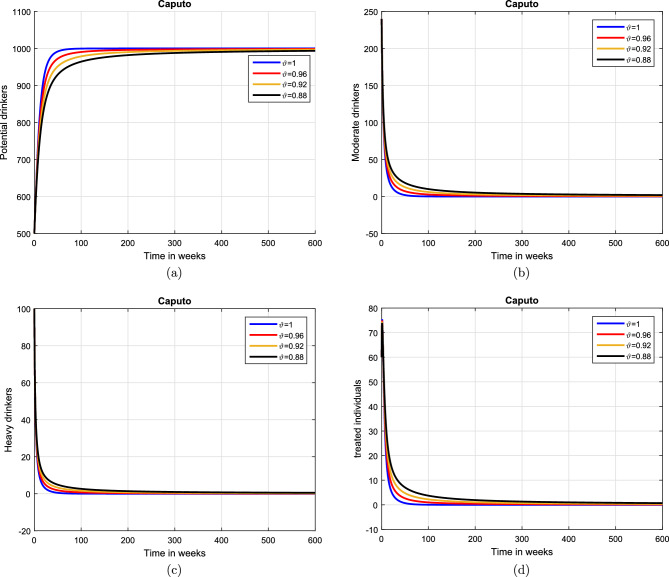
Dynamics of the (**a**) potential drinkers (**b**) moderate/occasional drinkers (**c**) heavy drinkers, (**d**) under treatment drinkers when $\mathcal {R}_{0}=0.870517<1$ and taking four values of fractional order $\vartheta$. These plots show the stability of AFE of the fractional alcohol addiction model (5).

**Figure 3:**
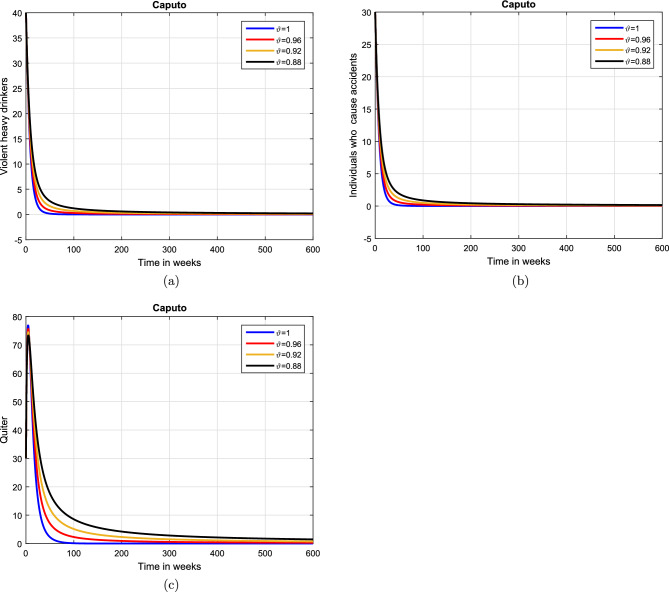
Dynamics of the (**a**) heavy drinkers creating violence (**b**) drinkers causing road accidents (**c**) Quitters/recoverd, when $\mathcal {R}_{0}=0.870517<1$ and taking four values of fractional order $\vartheta$. These plots show the stability of AFE of the fractional alcohol addiction model (5).

**Figure 4:**
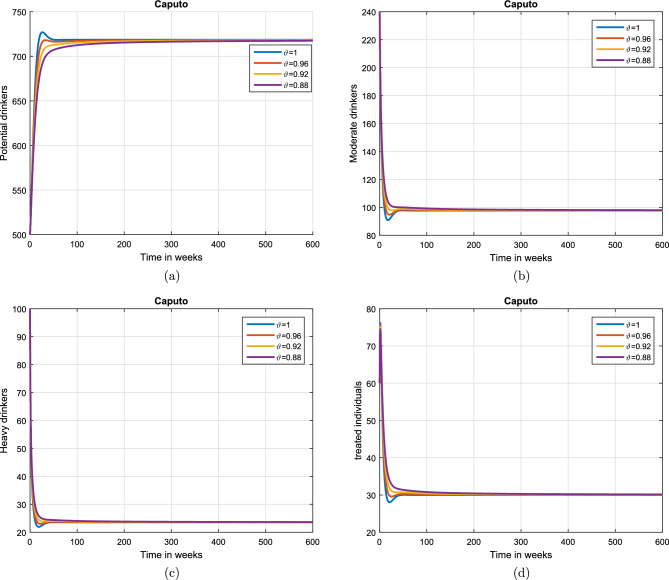
Simulation of the (**a**) potential drinkers (**b**) moderate/occasional drinkers (**c**) heavy drinkers, (**d**) under treatment drinkers when $\mathcal {R}_{0}=1.30577>1$ and taking four values of fractional order $\vartheta$. These plots demonstrate the stability of EE of the fractional alcohol addiction model (5).

**Figure 5:**
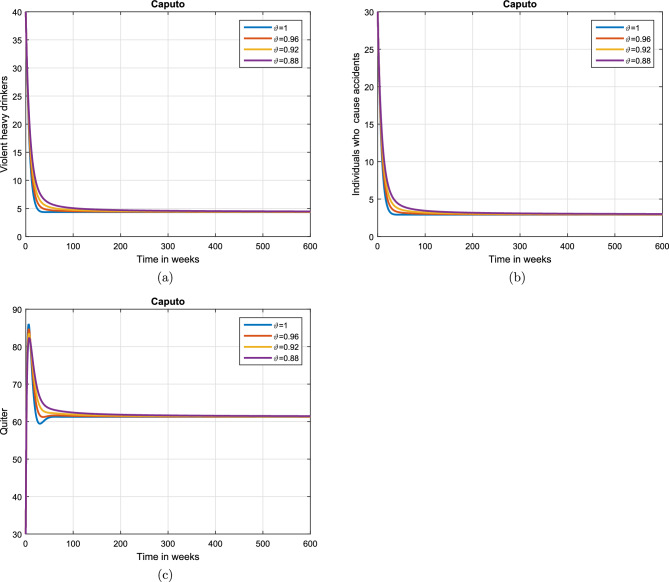
Simulation of the (**a**) heavy drinkers drinkers creating violence (**b**) drinkers causing road accidents (**c**) Quitters/recoverd, when $\mathcal {R}_{0}=1.30577>1$ and taking four values of fractional order $\vartheta$. These plots depict the stability of EE of the fractional alcohol addiction model (5).

- Case 1. We show the impact of FO of the Caputo derivative on the model’ solution in the first case. The parameters mentioned in Table 1 leads the value of $\mathcal {R}_{0}$ less than unity in the Figs. 2 and 3. It can be seen that for all fractional values $\vartheta$ the solution paths are convergent towards the AFE state.
- Case 2. Simulation in the second scenario is performed by enhancing the the parameter value $\beta _1$ to its baseline value so that the value of $\mathcal {R}_{0}$ is greater than 1. The population level dynamics are illustrated in Figs. 4 and 5. It can be seen that the solution paths are convergent towards the EE state. It is important to note that in both cases for greater values of $\vartheta$, the model solution converges more rapidly to steady states.

#### Impact of contact rates $\beta _{1}$, $\beta _{2}$ and FO$\vartheta$

**Figure 6:**
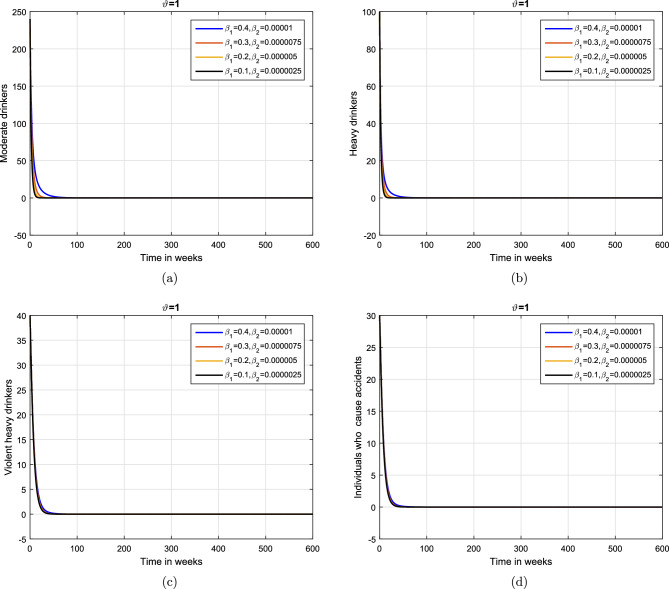
Impact of variation in contact rates $\beta _{1}$ and $\beta _{2}$ with $\vartheta =1.$.

**Figure 7:**
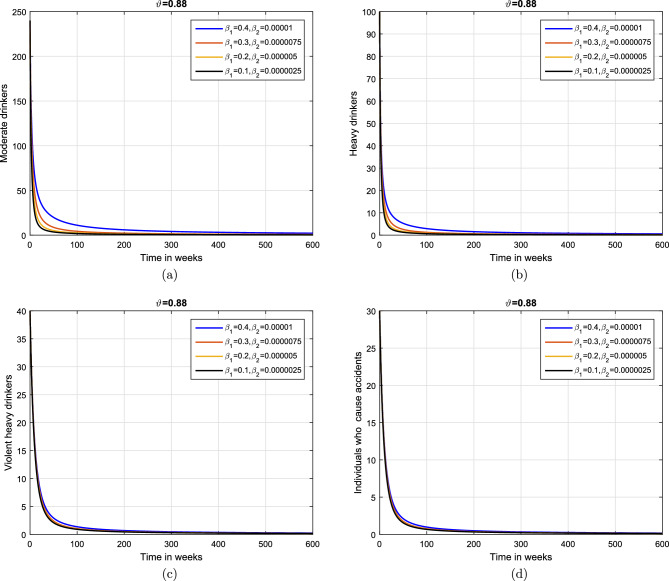
Impact of variation in contact rates $\beta _{1}$ and $\beta _{2}$ with $\vartheta =0.88.$.

**Figure 8:**
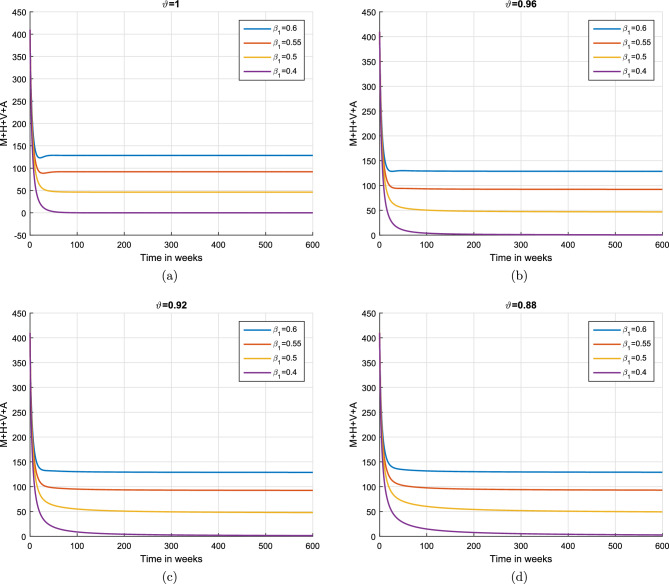
Impact of variation in contact rate $\beta _{1}$ on cumulative individuals in $\mathcal {M}$, $\mathcal {H}$, $\mathcal {V}$ and $\mathcal {A}$ groups for different values of $\vartheta .$.

This subsection explains the impact of the contact rates ($\beta _{1},\beta _{2}$) on moderate, heavy, violent heavy drinkers and individuals who cause accidents due to heavy drinking. Initially, the graphical results are produced using the parameter baseline values. After that, the parameter values are decreased by $25\%,\;50\%$, and $75\%$ to the given tabulated values. Furthermore, the simulation results are performed using two different fractional values. The graphical results for the moderate, heavy, violent heavy drinkers and individuals who cause accidents due to heavy drinking are depicted in Figs. 6 and 7. Additionally, it is clear from the Fig. 6 that fractional value $\vartheta =0.88$ gives biologically more significant insights of the dynamics of the disease. Figure 8 depict the impact of $\beta _{1}$ on cumulative classes using different fractional values while keeping value of $\beta _{2}$ fixed. It is noted that the population significantly declines as the effective contact rates decrease.

Based on the various numerical simulations presented, it is evident that the entire classes in the model eventually attain stability after a certain duration, and they all converge to a fixed population point. Furthermore, the variations in the transmission coefficient with different rates, a similar stable solution can be observed. From the biological perspective, such behaviors of the model revealed that alcohol addiction can be eradicated (or minimized) by implementing effective contorting interventions such as isolating, and proper medical, psychological, and social interventions of the addictive individuals.

## Alcohol addiction model in fractal–fractional perspective

Many real-life phenomena exhibit self-similar patterns. In epidemics, such behavior refers to the presence of repeated and similar structures at different time intervals within the spread of diseases. Understanding self-similar patterns in epidemics is essential for modeling and predicting future dynamics accurately. Moreover, it can help public health officials and researchers develop more effective preventive strategies for disease control. In addition, recognizing these patterns can aid in the early detection of emerging outbreaks and the optimization of healthcare resources. To address the scientific challenges posed by phenomena exhibiting self-replicating structures, novel differential and integral operators called fractal–fractional operators were introduced. We extend the fractional alcohol addiction model using the Caputo fractal–fractional operator defined in (2). The fractal–fractional compartmental model comprising the alcohol addiction behavior can be illustrated as follows

20 $$\begin{aligned} {}^{{FF}}D^{\vartheta , \zeta }_{0, t}\Big (\mathcal {P}(t)\Big )= & {} \Lambda - \frac{({\beta _1\mathcal {M}+\beta _2\mathcal {H}})\mathcal {P}}{\mathcal {N}} - \nu \mathcal {P},\nonumber \\ {}^{{FF}}D^{\vartheta , \zeta }_{0, t}\Big (\mathcal {M}(t)\Big )= & {} \frac{(\beta _1\mathcal {M}+\beta _2\mathcal {H})\mathcal {P}}{\mathcal {N}} - \left( {\psi + \nu } \right) \mathcal {M},\nonumber \\ {}^{{FF}}D^{\vartheta , \zeta }_{0, t}\Big (\mathcal {H}(t)\Big )= & {} \psi \mathcal {M} - \left( {\nu + \theta _1 + \theta _2 + \theta _3 + \delta _1 + \phi } \right) \mathcal {H}, \nonumber \\ {}^{{FF}}D^{\vartheta , \zeta }_{0, t}\Big (\mathcal {T}(t)\Big )= & {} \phi \mathcal {H} - \left( {\nu + \delta _2 + \gamma _1 } \right) \mathcal {T}, \nonumber \\ {}^{{FF}}D^{\vartheta , \zeta }_{0, t}\Big (\mathcal {V}(t)\Big )= & {} \theta _1 \mathcal {H} - \left( {\nu + \delta _3 + \gamma _2 } \right) \mathcal {V}, \nonumber \\ {}^{{FF}}D^{\vartheta , \zeta }_{0, t}\Big (\mathcal {A}(t)\Big )= & {} \theta _2 \mathcal {H}- \left( {\nu + \delta _4 + \gamma _3 } \right) \mathcal {A}, \nonumber \\ {}^{{FF}}D^{\vartheta , \zeta }_{0, t}\Big (\mathcal {Q}(t)\Big )= & {} \theta _3 \mathcal {H} + \gamma _1 \mathcal {T} + \gamma _2 \mathcal {V} + \gamma _3 \mathcal {A} - \nu \mathcal {Q}, \end{aligned}$$

under the assumption that the state variables are all positive, and with appropriate initial conditions. In the model (20), ${}^{{FF}}D^{\vartheta , \zeta }_{0, t}(.)$ is fractal–fractional Caputo operator where the fractional and fractal parameters are denoted as $\vartheta$ and $\zeta$ respectively. In the subsequent section, we will commence the fundamental analysis of this model.

### Application of fixed point theory to the alcohol addiction model

The existence and uniqueness of solutions for complex mathematical models can often be confirmed using fixed-point theory. This approach involves finding fixed points for certain mapping functions associated with model equations. When a unique fixed point is evaluated, it indicates the existence of a unique solution to the corresponding model. In this part of the study, we focused into establishing the existence and uniqueness of the solution for the proposed alcohol addiction model. This is accomplished by using the well-established Picard-Lindelof theorem in conjunction with the fixed-point approach. To proceeds, we can represent the Caputo fractal–fractional system (20) as a generalized Cauchy problem, which can be expressed as follows:

21 $$\begin{aligned} \left\{ \begin{array}{ll} {}^{{FF}} D^{\vartheta ,\zeta }_{0,t} g(t)=\mathcal {J}(t,g(t)),\\ g(0)=g_0, \;\; 0<t< \textit{T}<\infty , \end{array} \right. \end{aligned}$$

where *g*(*t*) is composed of state variables and $\mathcal {J}$ represents a continuous vector function defined in the right sides of the model (20). The corresponding initial values are specified by $g_0$.

The problem (21) yields the following outcome upon the utilization of the fractional integral:

22 $$\begin{aligned} \frac{1}{\Gamma (1-\vartheta )}\frac{d}{dt}\int ^t_0(t-r)^{-\vartheta } \mathcal {J}(t,g(t))dr=\zeta t^{\zeta -1}\mathcal {J}(t,g(t)). \end{aligned}$$

After substituting the right side with the Caputo operator and subsequently integrating, the resulting expression is as follows^43^:

23 $$\begin{aligned} g(t)=g_0+\frac{ \zeta }{\Gamma (\vartheta )}\int ^t_0 (t-r)^{\vartheta -1} r^{\zeta -1}\mathcal {J}(r,g(r))dr. \end{aligned}$$

Moreover, using the Picard Lindel$\ddot{o}$f approach, we give

$$\begin{aligned} \prod _a^b=\mathbb {I}_n(t_n)\times \overline{\mathbb {A}_0(p_0)}, \end{aligned}$$

where

$$\begin{aligned} \mathbb {I}_n(t_n)=[t_{n-a},t_{a+n}],\;and ;\overline{\mathbb {A}_0(p_0)}=[t_0+b,b+t_0]. \end{aligned}$$

Defining the following operator

$$\begin{aligned} \Psi .C[\mathbb {I}_n(t_n),\mathbb {A}_b(t_n)]\;\;into\;\; C(\mathbb {I}_n(t_n),\mathbb {A}_b(t_n)), \end{aligned}$$

where,

24 $$\begin{aligned} \Psi \phi (t)=g_0+\frac{ \zeta }{\Gamma (\vartheta )}\int ^t_0 (t-r)^{\vartheta -1}r^{\zeta -1}\mathcal {J}(r,\phi (r))dr. \end{aligned}$$

Our primary objective is to validate that the operator given in (24) maps a complete normed metric space into itself. Furthermore, it is essential to verify that this operator also satisfies the conditions of a contraction mapping. In our initial attempt, we aim to establish that:

25 $$\begin{aligned} \Vert \Psi \phi (t)-g_0\Vert \le c. \end{aligned}$$

We proceed by considering the norm defined as follows:

26 $$\begin{aligned} \Vert \Psi \phi (t)-g_0\Vert\le & {} \frac{ \zeta }{\Gamma (\vartheta )} \int ^t_0 (t-r)^{\vartheta }r^{\zeta -1}\Vert \mathcal {J}(r,\phi (r))\Vert _{\infty }dr \\\\\le & {} \frac{ \zeta }{\Gamma (\vartheta )} K \int ^t_0 (t-r)^{\vartheta }r^{\zeta -1}dr, \end{aligned}$$

where,

$$\begin{aligned} K=\Vert \mathcal {J}\Vert _{\infty }, \end{aligned}$$

and the norm is defined by

$$\begin{aligned} \Vert \chi \Vert _\infty =sup_{t\in \prod _a^b}\Vert \chi (t)\Vert . \end{aligned}$$

Additionally, by letting $r=ty$, the preceding expression is transformed into:

27 $$\begin{aligned}{} & {} \Vert \Lambda \phi (t)-g_0\Vert \le \frac{ \zeta K}{\Gamma (\vartheta )} t^{\vartheta +\zeta -1}B(\vartheta ,\zeta ), \end{aligned}$$

28 $$\begin{aligned}{} & {} \Vert \Lambda \phi (t)-g_0\Vert<c\Longrightarrow K<\frac{c\Gamma (\vartheta )}{ \zeta a^{\zeta +\vartheta -1}B(\zeta ,\vartheta )}. \end{aligned}$$

When considering $\phi _1$ and $\phi _2$ as continuous functions in the interval $[I_n(t_n), A_b(t_n)]$, we have derived the following outcome:

29 $$\begin{aligned} \Vert \Lambda \phi _1-\Lambda \phi _2\Vert\le & {} \Big (\frac{ \zeta L}{\Gamma (\vartheta )}t^{\zeta +\vartheta -1}B(\vartheta ,\zeta )\Big )\Vert \phi _1-\phi _2\Vert \nonumber \\< & {} \Big (\frac{ \zeta L}{\Gamma (\vartheta )}a^{\zeta +\vartheta -1}B(\vartheta ,\zeta )\Big )\Vert \phi _1-\phi _2\Vert . \end{aligned}$$

Hence, we conclude that the contraction criteria is achieved upon the fulfillment of the following condition.

30 $$\begin{aligned} L<\frac{\Gamma (\vartheta )}{ \zeta a^{\zeta +\vartheta -1}B(\vartheta ,\zeta )}. \end{aligned}$$

Hence, complete the proof.

### Numerical scheme for fractal–fractional alcohol addiction model

We provide a concise overview of a novel numerical scheme designed for the proposed transmission model in fractal–fractional perspective (20). The purpose of this scheme is to visualize the effects of fractional and fractal orders graphically in a simultaneous pattern. We adopt the procedure outlined in^43^. To begin, the system established in (20) is initially rewritten into Volterra integral since the fractional integral is differentiable. Subsequently, the fractal–fractional alcohol addition model in Riemann-Liouville sense is express as below

31 $$\begin{aligned} \frac{1}{\Gamma (1-\vartheta )}\frac{d}{dt}\int ^t_0(t-\zeta )^{-\vartheta } g(\zeta )d\zeta \frac{1}{\zeta t^{\zeta -1}}. \end{aligned}$$

The initial value problem stated in (21) can be converted as follows:

32 $$\begin{aligned} {}^{{RL}}D^{\vartheta }_{0, t} \Big (g(t)\Big )= & {} \zeta t^{\zeta -1}\Big [\mathcal {J}(t,g(t))\Big ], \end{aligned}$$

where

$$\begin{aligned} g(t)=\left( \begin{array}{ccccccc} \mathcal {P}(t)\\ \mathcal {M}(t)\\ \mathcal {H}(t)\\ \mathcal {T}(t)\\ \mathcal {V}(t)\\ \mathcal {A}(t)\\ \mathcal {Q}(t) \end{array} \right) ,~~ g(0)=\left( \begin{array}{ccccccc} \mathcal {P}(0)\\ \mathcal {M}(0)\\ \mathcal {H}(0)\\ \mathcal {T}(0)\\ \mathcal {V}(0)\\ \mathcal {A}(0)\\ \mathcal {Q}(0) \end{array} \right) , \end{aligned}$$

and

$$\begin{aligned} \mathcal {J}(t,g(t))=\left( \begin{array}{ccccccc} \mathcal {J}_1(t,g(t))\\ \mathcal {J}_2(t,g(t))\\ \mathcal {J}_3(t,g(t))\\ \mathcal {J}_4(t,g(t))\\ \mathcal {J}_5(t,g(t))\\ \mathcal {J}_6(t,g(t))\\ \mathcal {J}_7(t,g(t)) \end{array} \right) =\left( \begin{array}{cccccccc} \Lambda - \frac{({\beta _1\mathcal {M}+\beta _2\mathcal {H}})\mathcal {P}}{\mathcal {N}} - \nu \mathcal {P}\\ \frac{(\beta _1\mathcal {M}+\beta _2\mathcal {H})\mathcal {P}}{\mathcal {N}} - \left( {\psi + \nu } \right) \mathcal {M}\\ \psi \mathcal {M} - \left( {\nu + \theta _1 + \theta _2 + \theta _3 + \delta _1 + \phi } \right) \mathcal {H}\\ \phi \mathcal {H} - \left( {\nu + \delta _2 + \gamma _1 } \right) \mathcal {T}\\ theta _1 \mathcal {H} - \left( {\nu + \delta _3 + \gamma _2 } \right) \mathcal {V}\\ \theta _2 \mathcal {H}- \left( {\nu + \delta _4 + \gamma _3 } \right) \mathcal {A}\\ \theta _3 \mathcal {H} + \gamma _1 \mathcal {T} + \gamma _2 \mathcal {V} + \gamma _3 \mathcal {A} - \nu \mathcal {Q} \end{array} \right) . \end{aligned}$$

Furthermore, we replace the derivative in Riemann-Liouville sense by the Caputo case to facilitate the utilization of integer-order initial values. The subsequent results are then obtained by applying the Riemann-Liouville fractional integral to both sides:

33 $$\begin{aligned} g(t)= & {} g_0+\frac{\zeta }{\Gamma (\vartheta )}\int ^t_0 r^{\zeta -1}(t-r)^{\vartheta -1}\mathcal {J}(r,g(r))d r. \end{aligned}$$

At $t=t_{n+1}$, we have from (33)

34 $$\begin{aligned} g(t_{n+1})= & {} g_0+\frac{\zeta }{\Gamma (\vartheta )}\int ^{t_{n+1}}_0 r^{\zeta -1}(t_{n+1}-r)^{\vartheta -1}\mathcal {J}(r,g(r))d r,\nonumber \\= & {} g_0+\frac{\zeta }{\Gamma (\vartheta )}\sum ^{n}_{v=0}\int ^{t_{v+1}}_{t_v} r^{\zeta -1}(t_{n+1}-r)^{\vartheta -1}\mathcal {J}(r,g(r))d r. \end{aligned}$$

Furthermore, by employing the well-known Lagrangian piece-wise interpolation approach upon $[t_v, t_{v+1}]$ for $\mathcal {J}(r,g(r))$ in (34), we obtain:

35 $$\begin{aligned} \mathcal {J}(r,g(r))\approx g_\ell (r)= & {} \frac{r-t_{\ell -1}}{t_\ell -t_{\ell -1}}t^{\zeta -1}_\ell \mathcal {J}(t_\ell ,g(t_\ell ))-\frac{r-t_{\ell }}{t_\ell -t_{\ell -1}}t^{\zeta -1}_{\ell -1}\mathcal {J}(t_{\ell -1},g(t_{\ell -1})). \end{aligned}$$

By employing the approximation outlined in (35), equation (34) results in the following:

36 $$\begin{aligned} g(t_{n+1})= & {} g_0+\frac{\zeta }{\Gamma (\vartheta )}\sum ^{n}_{\ell =0}\int ^{t_{\ell +1}}_{t_\ell } r^{\zeta -1}(t_{n+1}-r)^{\vartheta -1} g_\ell (r)d r. \end{aligned}$$

In a result of solving (36), the following iterative scheme is derived

37 $$\begin{aligned} g(t_{n+1})= & {} g_0+\frac{\zeta {\mathfrak {h}}^{\vartheta }}{\Gamma (\vartheta +2)}\sum ^n_{\ell =0} \Big [t^{\zeta -1}_\ell \mathcal {J}(t_\ell ,g(t_\ell ))\nonumber \\{} & {} \times \Big ((n+1-\ell )^{\vartheta }(n-\ell +2+\vartheta )-(n-\ell )^{\vartheta }(n+2+2\vartheta -\ell )\Big )\nonumber \\{} & {} -t^{\zeta -1}_{\ell -1}\mathcal {J}(t_{\ell -1},g(t_{\ell -1})) \times \Big ((n-\ell +1)^{\vartheta +1}-(n-\ell )^{\vartheta }(n-\ell +1+\vartheta )\Big )\Big ]. \end{aligned}$$

### Simulation of the alcohol addiction model with fractal–fractional operator

**Figure 9:**
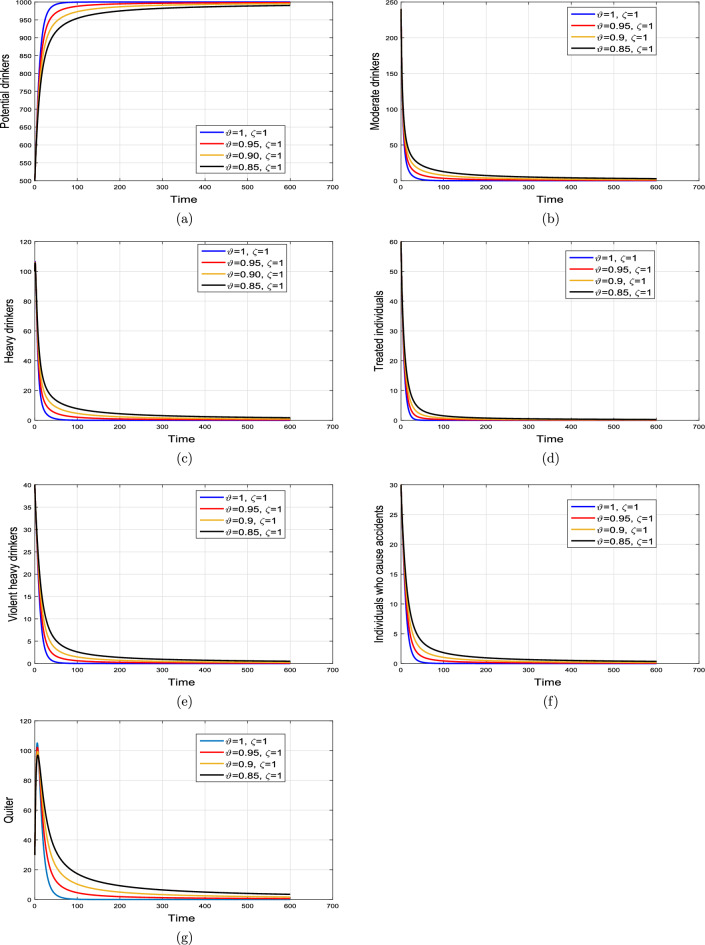
Visual analysis of the Caputo fractional-fractal alcohol addiction compartmental model (20) by considering $\vartheta =0.85,0.90,0.95,1$, $\zeta =1$ and $\mathcal {R}_0<1$. These plots show the stability of AFE of the model (20).

**Figure 10:**
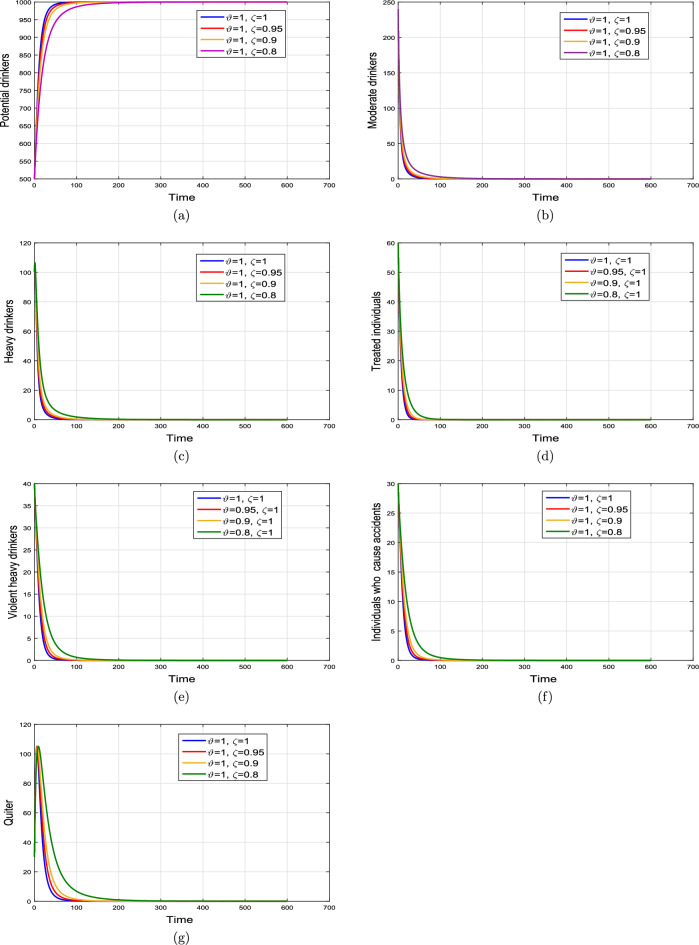
Visual analysis of the Caputo fractional-fractal alcohol addiction compartmental model (20) by considering $\vartheta =1$, $\zeta =0.80,0.90,0.95,1$ and $\mathcal {R}_0<1$. These plots demonstrate the stability of AFE of the fractal–fractional model (20).

**Figure 11:**
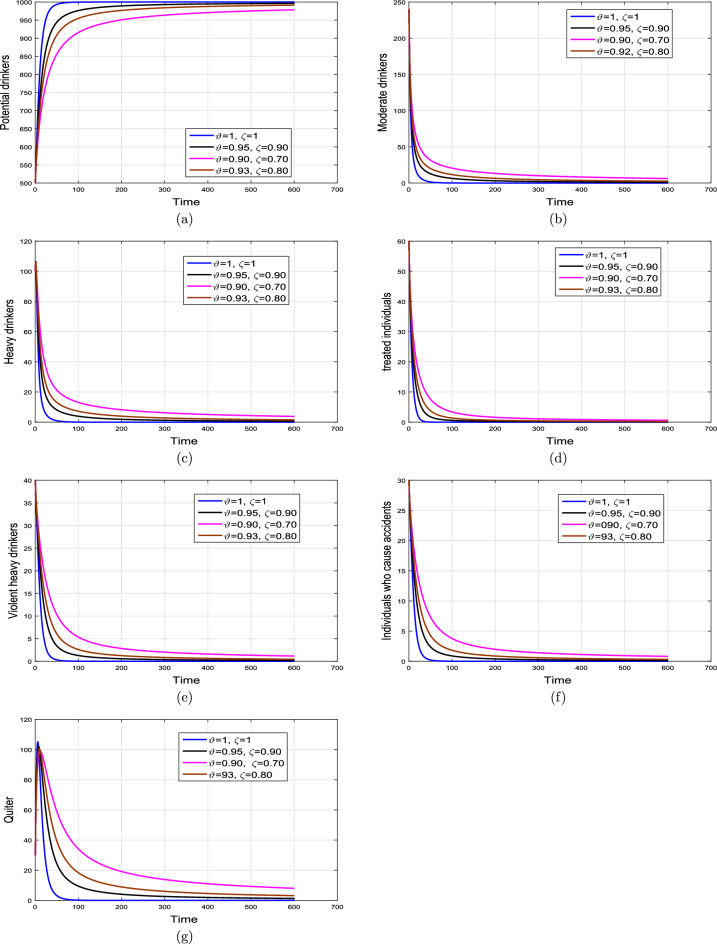
Visual analysis of the Caputo fractional-fractal alcohol addiction compartmental model (20) by considering $\vartheta =1,0.95,0.90,0.85$, $\zeta =1,0.90,0.70,0.80$ and $\mathcal {R}_0<1$. These plots depict the stability of AFE of the fractal–fractional model (20).

The simulation of the alcohol addiction model in fractal–fractional case (20) is performed using the numerical scheme derived in (37) for the cases when $\mathcal {R}_0<1$ and $\mathcal {R}_0>1$. The parameter values and ICs remain the same with those taken in only the fractional case model. We conduct simulations for three scenarios of both above two cases, each involving distinct values of the fractal order $\zeta$ and fractional order $\vartheta$. The numerical analysis of the model simulations is presented in Figs. 9, 10, 11. In all plots, we depict the influence of the fractal and fractional orders, $\vartheta$ and $\zeta$, respectively, on the model’s solution. The detailed discussion on each case is as follows:

#### Visual dynamics of the model when $\mathcal {R}_0<1$

*Case 1:* In this case, we illustrate the dynamics of the alcohol addiction model when the fractal dimension $\zeta$ remains fixed to the integer value and taking four different values of the fractional order $\vartheta$ i.e.,$\vartheta =0.85,0.90,0.95,1$ and $\zeta =1$. The parameters given in Table 1 are used for the simulation of this case whereas the resulting visual dynamics are shown in Fig. 9a–g. For this set of parameters, we have $\mathcal {R}_0=0.2731<1$ and therefore we can see that the solution curves converge to the AFE $\mathcal {D}^{0}$ for all fractional and fractal dimensions.

*Case 2:* This scenario illustrates the visual dynamical aspects of the alcohol addiction model in a fractal–fractional case (20) when the fractional dimension $\vartheta$ remains fixed to the integer value and considering four different values of the fractional dimension $\zeta$ i.e.,$\zeta =0.80,0.90,0.95,1$ and $\vartheta =1$. The simulation of this case comprising the visual dynamics of the alcohol addiction model is shown in Fig. 10a–g. One can observe a similar behavior of the model solution seen in Case 1 i.e., AFE $\mathcal {D}^{0}$ is globally asymptotically stable for values of $\zeta$ and $\vartheta$.

*Case 3:* In the third scenario we demonstrate the impact when both the fractional $\vartheta$ and fractal dimension $\zeta$ are varied simultaneously i.e., $\vartheta =1,0.95,0.90,0.85$ and $\zeta =1,0.90,0.70,0.80$. We analyze the simulation results obtained for this scenario in Fig. 11a–g. Similar to the previous cases, the solution curves converge to the AFE $\mathcal {D}^{0}$ for all fractional and fractal dimensions. However, it can be seen in this case that for smaller values of the fractional and fractal orders, the solution tends to the equilibrium point comparatively over a longer time interval.

We observed biological more feasible results of the model at higher fractal–fractional orders. The simulation of the fractal–fractional alcohol transmission model (20) reveals that the solution curves in all population classes tend to converge rapidly to a stable scenario at higher fractal–fractional orders, while the convergence is slower at lower fractal–fractional orders. Ultimately, the model quickly attains the AFE. Furthermore, it is evident that as the fractal–fractional orders increase, the density of potential drinkers also rises. On the other hand, the density of the remaining population groups decreases with an increase in the fractal–fractional orders.

#### Dynamics of the model when $\mathcal {R}_0>1$

In this subpart, we continue with the simulation of the Caputo fractional-fractal alcohol addiction compartmental model (20) for the case when $\mathcal {R}_0>1.$ Some of the parameters are perturbed such that the basic reproductive number exceeds unity. In a similar way to the previous section, we discuss the simulation of this scenario in the following three sub-cases.

**Figure 12:**
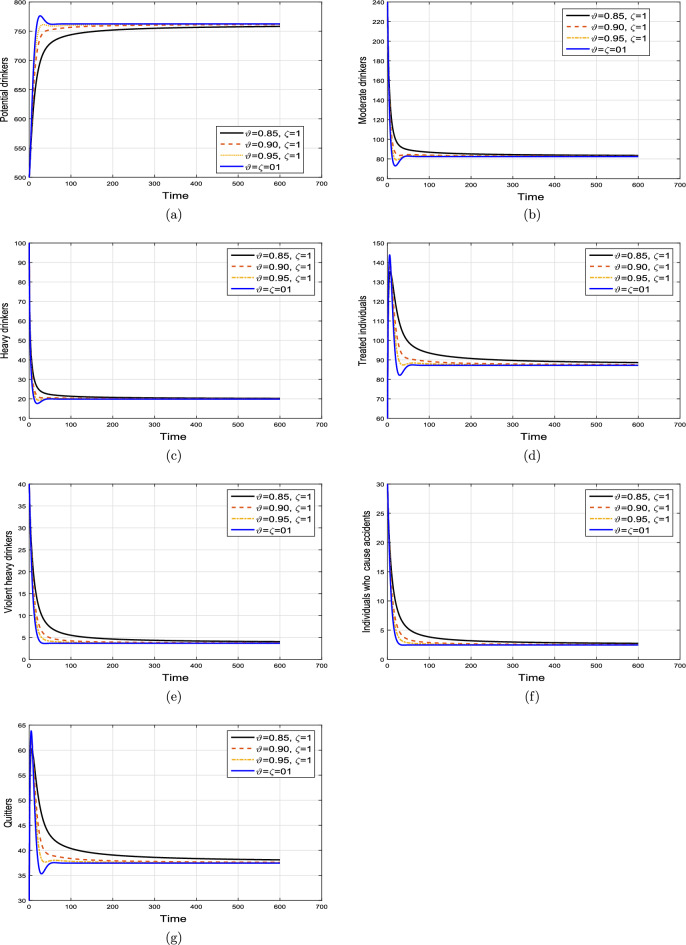
Visual analysis of the Caputo fractional-fractal alcohol addiction compartmental model (20) by considering $\vartheta =1,0.95,0.90,0.80$, $\zeta =1$ and $\mathcal {R}_0>1$. These plots demonstrate the stability of EE of the fractal–fractional model (20).

*Case 1:* In the first case, the fractal–fractional model (20) is simulated by taking four different values of the fractional order $\vartheta$ i.e.,$\vartheta =0.80,0.90,0.95,1$ and keeping the fractal order fixed as $\zeta =1$. The visual dynamics of the model of various population classes are shown in Fig. 12a–g. Since, the basic reproductive number $\mathcal {R}_0>1$ in this case therefore, we can see that the solution curves converge to the EE $\mathcal {E}^{*}$ for all values of fractal and fractional operators.

**Figure 13:**
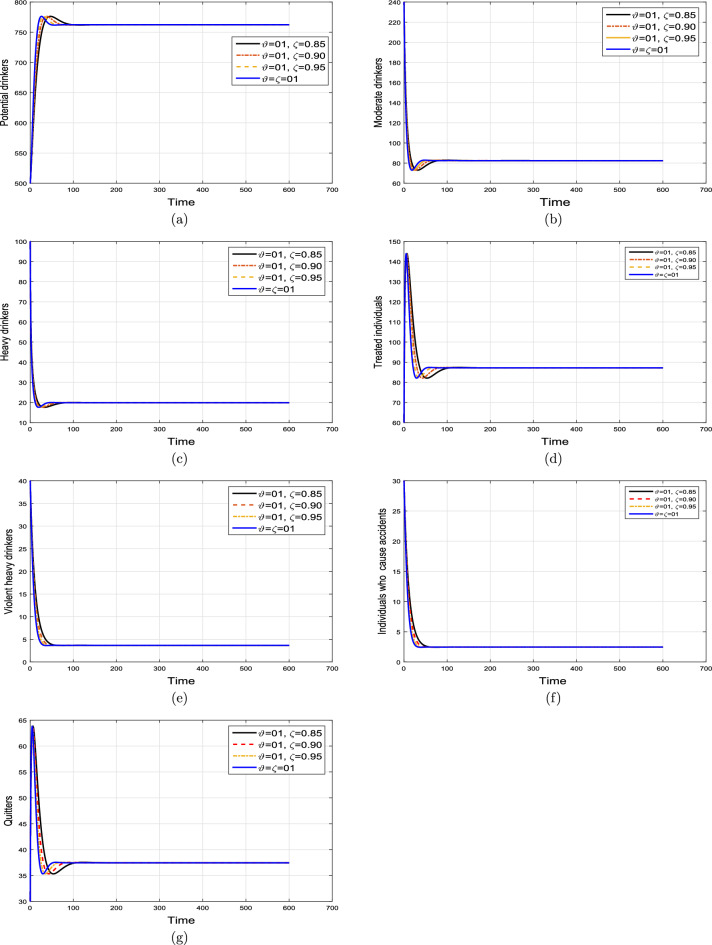
Visual analysis of the Caputo fractional-fractal alcohol addiction compartmental model (20) by considering $\vartheta =1$ and $\zeta =1,0.95,0.90,0.85$ and $\mathcal {R}_0>1$. These plots show the stability of EE of the fractal–fractional model (20).

*Case 2:* In the second case, the parameters are set in a way that $\mathcal {R}_0>1$, and the model is simulated when the fractional dimension $\vartheta$ is considered to be the integer value and the fractional dimension $\zeta$ is varied such that $\zeta =0.85,0.90,0.95,1$. The graphical behavior of model’s solution under this case is depicted in Fig. 13a–g revealing the convergence of the EE $\mathcal {E}^{*}$.

**Figure 14:**
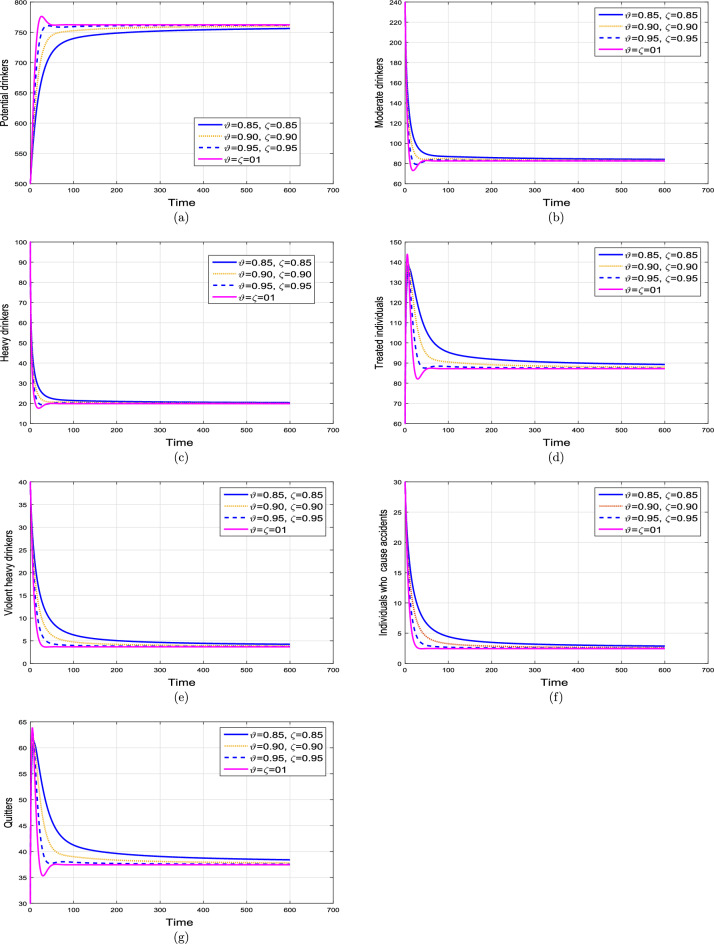
Visual analysis of the Caputo fractional-fractal alcohol addiction compartmental model (20) by considering $\vartheta =1,0.95,0.90,0.85$ and $\zeta =1,0.95,0.90,0.85$ and $\mathcal {R}_0>1$. These plots demonstrate the stability of EE of the fractal–fractional model (20).

*Case 3:* Finally, in this case, the addiction fractional-fractal is simulated when both the fractional $\vartheta$ and fractal dimension $\zeta$ are varied simultaneously i.e., $\vartheta =1,0.95,0.90,0.85$ and $\zeta =1,0.95,0.90,0.85$. The visual dynamics of model’s compartments are analyzed in Fig. 14a–g. Similar to the previous cases, the solution curves converge to the EE $\mathcal {E}^{*}$ for all fractional and fractal dimensions.

Similar to the scenario when $\mathcal {R}_0<1$, the simulation of this case demonstrates that the solution graphs in population groups converge quickly to a stable equilibrium at higher fractal–fractional orders, whereas the convergence is slower at lower fractal–fractional orders. Furthermore, at higher fractal–fractional orders, there is an increase in the density of potential drinkers, while the density of the remaining population groups decreases.

## Conclusion

In this study, we established a new mathematical model analyzing the dynamics of alcohol addiction behavior, one of the recent major health and economic concerns. Unlike the existing literature, the proposed model is developed using the well-established fractional and fractal-factional operators under the power-law. The population is classified into seven different subclasses to formulate the alcohol addiction model. The models in both cases are rigorously analyzed from a qualitative point of view. To examine the qualitative feature of solutions, the existence and uniqueness criterions for the solution of the fractal–fractional model using well-known fixed point theorems. The necessary components of the models including possible equilibrium points and the threshold parameter are evaluated. In addition, the stability analysis was investigated using the Ulam–Hyers as well as the Ulam–Hyers–Rassias stability approaches. The visual global dynamics of the fractional and fractal–fractional addiction model are analyzed whenever the basic reproduction is less than and greater than unity. We concluded that for $\mathcal {R}_0<1$, the model solutions converge rapidly to alcohol-free equilibrium at higher fractal–fractional orders, while the convergence is slower at lower fractal–fractional orders. Thus, the alcohol addiction can be control in the community. On the other hand, for $\mathcal {R}_0>1$, the model solutions converge to endemic steady states for all values of fractal and fractional parameters and the addiction will persist. In particular, we observed biological more feasible results of the model at higher fractional and fractal–fractional orders as all population classes in the model reach to a stability after a certain time period and all converge to a fixed point of the population. Moreover, the impact of memory index and variation in some controlling parameters are shown on the eradication of alcohol addiction. In future, the dynamics of the proposed alcohol addiction model can be studied under the fractional operators with nonlocal and nonsingular kernel. Moreover, we will extend the alcohol addiction model by incorporating time-varying control interventions.

## Data Availability

The data that support the findings of this study are available from the corresponding author upon reasonable request. Further, no experiments on humans and/or the use of human tissue samples involved in this study.
